# Proactive vaccination using multiviral Quartet Nanocages to elicit broad anti-coronavirus responses

**DOI:** 10.1038/s41565-024-01655-9

**Published:** 2024-05-06

**Authors:** Rory A. Hills, Tiong Kit Tan, Alexander A. Cohen, Jennifer R. Keeffe, Anthony H. Keeble, Priyanthi N. P. Gnanapragasam, Kaya N. Storm, Annie V. Rorick, Anthony P. West, Michelle L. Hill, Sai Liu, Javier Gilbert-Jaramillo, Madeeha Afzal, Amy Napier, Gabrielle Admans, William S. James, Pamela J. Bjorkman, Alain R. Townsend, Mark R. Howarth

**Affiliations:** 1 Department of Biochemistry, University of Oxford, Oxford, UK; 2 Department of Pharmacology, University of Cambridge, Cambridge, UK; 3 MRC Human Immunology Unit, MRC Weatherall Institute of Molecular Medicine, John Radcliffe Hospital, University of Oxford, Oxford, UK; 4 Division of Biology and Biological Engineering, California Institute of Technology, Pasadena, CA USA; 5 James & Lillian Martin Centre, Sir William Dunn School of Pathology, University of Oxford, Oxford, UK; 6 Centre for Translational Immunology, Chinese Academy of Medical Sciences Oxford Institute, University of Oxford, Oxford, UK

**Keywords:** Nanoparticles, SARS-CoV-2

## Abstract

Defending against future pandemics requires vaccine platforms that protect across a range of related pathogens. Nanoscale patterning can be used to address this issue. Here, we produce quartets of linked receptor-binding domains (RBDs) from a panel of SARS-like betacoronaviruses, coupled to a computationally designed nanocage through SpyTag/SpyCatcher links. These Quartet Nanocages, possessing a branched morphology, induce a high level of neutralizing antibodies against several different coronaviruses, including against viruses not represented in the vaccine. Equivalent antibody responses are raised to RBDs close to the nanocage or at the tips of the nanoparticle’s branches. In animals primed with SARS-CoV-2 Spike, boost immunizations with Quartet Nanocages increase the strength and breadth of an otherwise narrow immune response. A Quartet Nanocage including the Omicron XBB.1.5 ‘Kraken’ RBD induced antibodies with binding to a broad range of sarbecoviruses, as well as neutralizing activity against this variant of concern. Quartet nanocages are a nanomedicine approach with potential to confer heterotypic protection against emergent zoonotic pathogens and facilitate proactive pandemic protection.

## Abstract (web summary)

The ability to vaccinate against multiple related pathogens is a significant advantage. Here, the authors report on quartets of linked receptor-binding domains attached to designed nanocages using SpyTag/SpyCatcher links, demonstrating effective vaccination against similar viruses as well as the variant of concern.

## Main

Nanoscale organization is a key signal for the programming of immune responses^1–3^. Highly multivalent display of antigens on virus-like particles (VLPs) or other nanoparticles enhances the strength and persistence of immune responses, facilitating lymph node uptake and increasing B cell receptor (BCR) clustering^1,2^. VLP manufacturing uses existing facilities for microbial fermentation to facilitate large-scale production^4^ and can avoid the need for a cold-chain^5^, and VLPs have shown a good balance of safety and efficacy^6^.

Existing vaccination strategies have shown success in reducing death and serious illness from SARS-CoV-2 (SARS2)^7^. Nevertheless, waning vaccine protection, continuing emergence of new variants and uncertain efficacy of therapeutics mean that new vaccine strategies are still urgently needed^8,9^. It is also important to protect against new pandemic threats from coronaviruses, which previously led to SARS-CoV (SARS1) and MERS-CoV outbreaks^10^. Other zoonotic coronaviruses such as WIV1 and SHC014 have been identified as having pandemic potential^11^. Immunizing with a single antigen typically induces a narrow strain-specific immune response, which may not protect against diverse pre-existing strains or newly arising variants of that pathogen^12^.

**Fig. 1:**
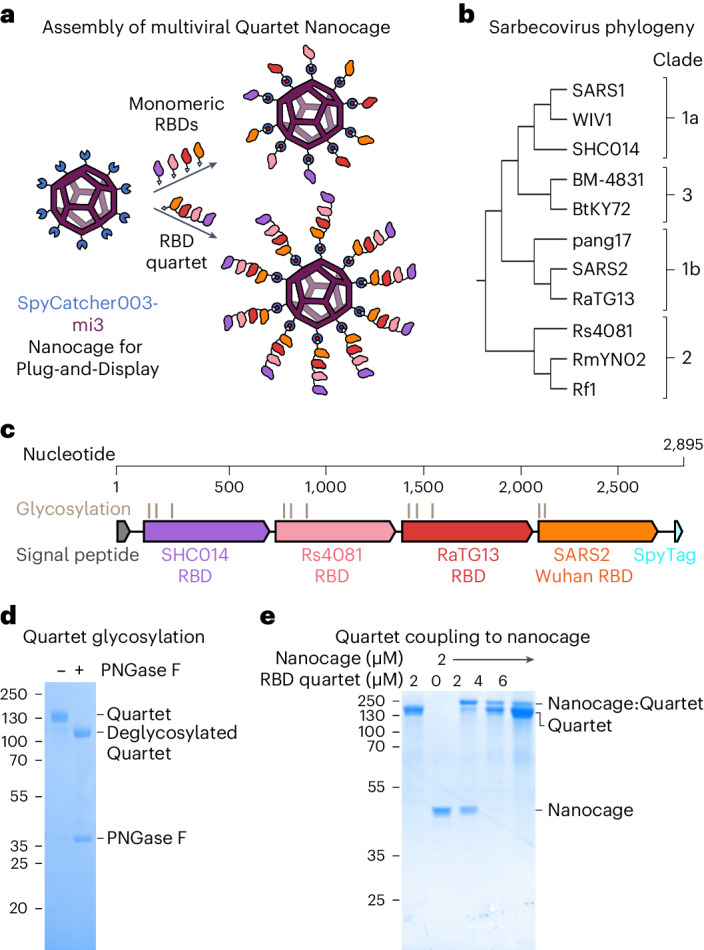
Preparation of multiviral Quartet Nanocages. **a**, Plug-and-Display vaccine assembly of mosaic and Quartet Nanocages. Genetic fusion of SpyCatcher003 (dark blue) with mi3 (purple) allows efficient multimerization of single or Quartet RBDs linked to SpyTag (cyan) through spontaneous isopeptide bond formation (marked in red). Only some antigens are shown in the schematic for clarity. **b**, Phylogenetic tree of sarbecoviruses used in this study, based on RBD sequence. **c**, Genetic organization of the multiviral Quartet-SpyTag, indicating the viral origin of RBDs, *N*-linked glycosylation sites and tag location. **d**, Analysis of Quartet-SpyTag with SDS–PAGE/Coomassie staining, with or without PNGase F deglycosylation. A representative gel from two independent experiments. Molecular weight markers are in kDa. **e**, Coupling of RBD Quartet to SpyCatcher003-mi3 Nanocage at different molar Nanocage:antigen ratios, analysed by SDS–PAGE/Coomassie. A representative gel from two independent experiments. Molecular weight markers are in kDa. Source data

In a recently introduced approach, VLPs display a panel of protein variants arranged stochastically on their surface, to drive expansion of B cells recognizing common features of the different antigens. A mosaic of different hemagglutinin heads on ferritin nanoparticles elicited cross-reactive antibodies against diverse influenza strains within the H1 subtype^13^. This approach has been applied to SARS2, with mosaic nanoparticles displaying multiple RBDs from the Spike of different sarbecoviruses^12,14,15^. Sarbecoviruses are the subgenus of betacoronaviruses that includes SARS1 and SARS2. RBDs can be multimerized on VLPs through genetic fusion^15^ or isopeptide coupling^12^. Fusion of a set of sarbecovirus RBDs with SpyTag003 facilitates simple nanoassembly onto the SpyCatcher003-mi3 VLP^12^ (Fig. 1a). SpyCatcher003 is a protein that we engineered to rapidly form an isopeptide bond with SpyTag peptide^16^. mi3 is a 60-mer hollow protein nanocage, computationally designed to self-assemble into a stable dodecahedron^17,18^.

In our previous study, the broadest immune response came from mosaic particles displaying eight different RBDs^12,14^. These Mosaic-8 nanoparticles elicited neutralizing antibodies against a variety of sarbecoviruses in mouse and rhesus macaque models. Critically, responses were not limited to viruses whose RBDs were represented on Mosaic-8 nanoparticles and included mismatched responses against heterologous sarbecoviruses^12,14^. Mosaic-8 nanoparticles have gained support from the Coalition for Epidemic Preparedness Innovations to enter clinical trials. However, the need to produce nine different components (eight RBDs and SpyCatcher003-mi3) at Good Manufacturing Practice level creates a challenge for broad scaling.

Here, we establish the production of multiviral Quartet Nanocages (Fig. 1a). Initially we express a multiviral Quartet from RBDs of four different viruses, concatenated as a single polypeptide chain. These antigenic Quartets are assembled via a terminal SpyTag to extend out from SpyCatcher003-mi3 nanocages, creating a protein nanoparticle with a branched morphology. This nanoassembly route reduces the number of vaccine components, as well as creating an architecture that allows a greater number of RBDs to be displayed on each nanocage. We measure antibody responses to the range of sarbecoviruses displayed on the Quartet Nanocage, to sarbecoviruses not present within the chain, as well as to SARS2 variants of concern (VOCs). Comparing different nanoassemblies, we dissect the breadth of antibody binding to different sarbecoviruses, neutralization potency and the ability to boost a broad response following focused priming. The magnitude and breadth of antibody induction show that Quartet Nanocages may provide a scalable route to induce neutralizing antibodies across a range of related viruses, to prepare for emerging outbreak disease threats.

## Design of multiviral Quartet Nanocages

The SARS2 RBD is directly involved in binding to the cell receptor angiotensin-converting enzyme 2 (ACE2) and is the target of most neutralizing antibodies^15^. We genetically fused RBDs from the evolutionarily related sarbecoviruses SHC014, Rs4081, RaTG13 and SARS2 Wuhan (Fig. 1b and Supplementary Figs. 1 and 2) to produce a multiviral Quartet (Fig. 1c). These RBDs allow comparison with the previously described Mosaic-4 vaccine^12^. The multiviral Quartet was engineered with a signal sequence for secretion from mammalian cells and a terminal SpyTag, to enable multivalent display on SpyCatcher003-mi3 (Fig. 1a). The Quartet was secreted efficiently by Expi293F cells and affinity-purified via SpyTag using the SpySwitch system^19^ (Supplementary Fig. 3). The Quartet band was broad on SDS–PAGE because of natural variation in glycosylation (Fig. 1d). Removing N-linked glycans with peptide N-Glycosidase F (PNGase F) induced a downward shift on the gel (Fig. 1d). Quartet-SpyTag gave a uniform peak by size-exclusion chromatography (Supplementary Fig. 4a). We demonstrated that the Quartet coupled efficiently to SpyCatcher003-mi3 (Fig. 1e).

## Quartet Nanocages raise antibodies to diverse sarbecoviruses

**Fig. 2:**
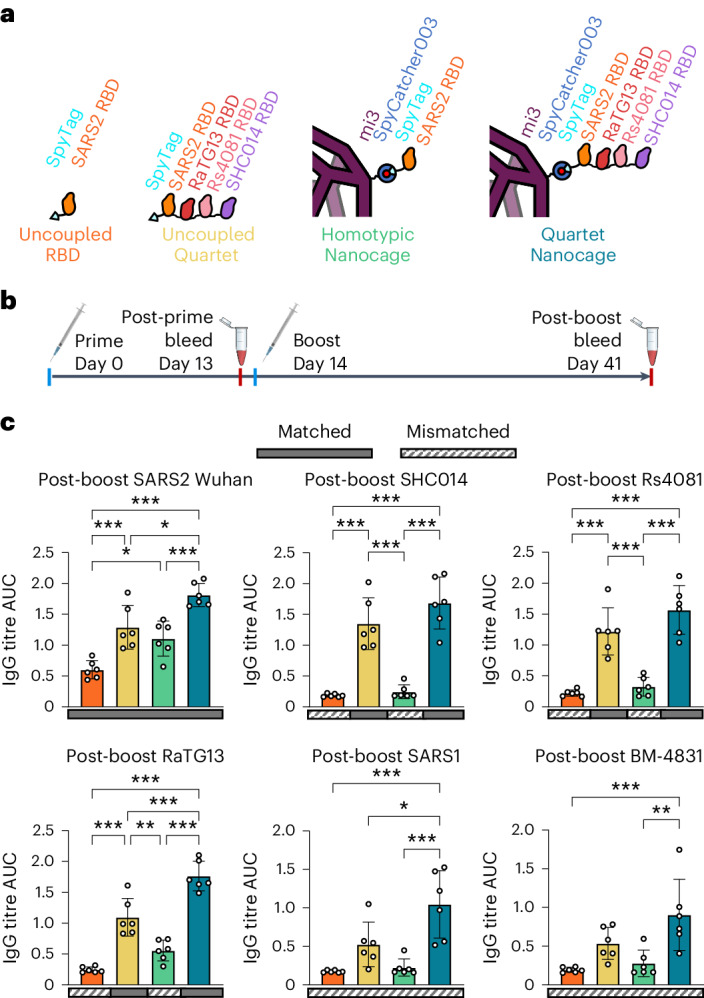
Broad immune response from immunization with Quartet Nanocages. **a**, Schematic of antigens for this set of immunizations, comparing uncoupled proteins or proteins coupled to the SpyCatcher003-mi3 nanocage. **b**, Procedure for immunization and sampling. **c**, ELISA for post-boost serum IgG binding to different sarbecovirus RBDs is shown as the area under the curve (AUC) of a serial dilution. Sera are from mice immunized with uncoupled SARS2 Wuhan RBD (orange), uncoupled Quartet-SpyTag (yellow), SARS2 Wuhan RBD coupled to SpyCatcher003-mi3 (green) or Quartet-SpyTag coupled to SpyCatcher003-mi3 (blue). Solid rectangles under samples indicate ELISA against a component of that vaccine (matched). Striped rectangles indicate ELISA against an antigen absent in that vaccine (mismatched). Each dot represents one animal. The mean is denoted by a bar, shown ±1 s.d.; *n* = 6. Significance was calculated with an ANOVA test using Tukey’s post hoc test. *P < 0.05, **P < 0.01, ***P < 0.001; other comparisons were non-significant. Source data

We then explored the Quartet’s immunogenicity as a soluble protein or multimerized on nanocages (Fig. 2a). Doses for all immunizations were normalized by the number of SpyTags, allowing comparison of a molar equivalent of SpyCatcher003-mi3 nanocages with similar levels of occupancy. Two doses were administered to mice 14 d apart using alum-based adjuvant (Fig. 2b), before quantifying IgG titre against RBD antigens by enzyme-linked immunosorbent assay (ELISA). Post-prime, the Quartet Nanocage elicited the highest antibody titre against SARS2 Wuhan RBD, surpassing the Homotypic Nanocage and Uncoupled Quartet (Supplementary Fig. 5a). The Quartet Nanocage also elicited a strong post-prime response to SARS1 RBD, not represented on the immunogen (Supplementary Fig. 5a), with a titre greater than the response against SARS2 Wuhan by Homotypic Nanocage (Supplementary Fig. 5).

After boosting, we similarly found the strongest response against SARS2 from Quartet Nanocage, followed by Uncoupled Quartet, Homotypic Nanocage and finally Uncoupled RBD (Fig. 2c and Supplementary Figs. 6 and 7). This pattern is retained for SARS2 Wuhan, Beta and Delta Spikes (Supplementary Fig. 5b). Uncoupled RBD and Homotypic Nanocage immunization raised low responses against the panel of sarbecovirus RBDs, with the greatest Homotypic Nanocage cross-reactive response against the closely related RaTG13 RBD (Fig. 2c). In contrast, we saw substantial responses against all tested RBDs with Uncoupled Quartet and most substantially Quartet Nanocage immunization (Fig. 2c). This included a strong heterotypic response against BM-4831 and SARS1 RBDs, which were absent from the chain and elicited titres only slightly lower than Homotypic Nanocage against SARS2 Wuhan (Fig. 2c). These results suggest the potential of this Quartet Nanocage approach to induce antibodies against a broad range of sarbecoviruses. We had hypothesized that RBDs at the tip of the Quartet would give stronger responses than RBDs nearer the nanocage surface. In fact, we saw no obvious relationship between RBD chain location and antibody titre (Fig. 2c).

## Comparison of Quartet Nanocages and Mosaic nanoparticles

**Fig. 3:**
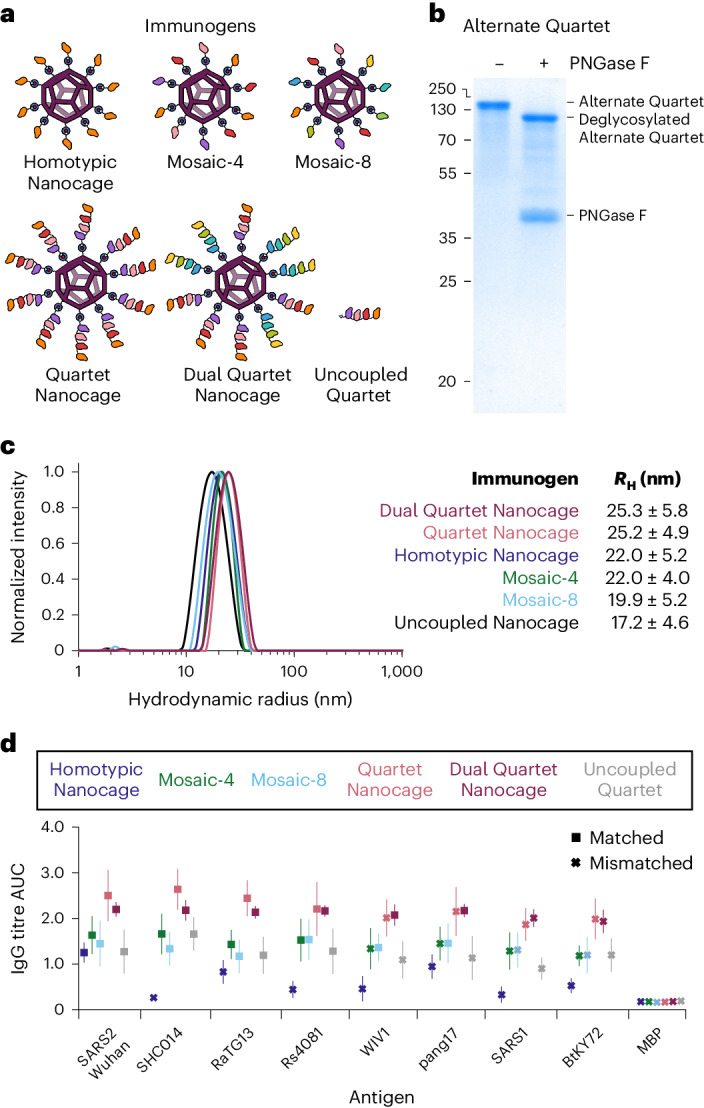
Comparison of immunization with Mosaic or Quartet Nanocages. **a**, Schematic of antigens for this set of immunizations. **b**, Validation of the Alternate Quartet by SDS–PAGE with Coomassie staining, shown ±PNGase F deglycosylation. A representative gel from two independent experiments. Molecular weight markers are in kDa. **c**, DLS of SpyCatcher003-mi3 alone (uncoupled nanocage) or each immunogen. The mean hydrodynamic radius (*R*_H_) is shown ±1 s.d., derived from 20 scans of the sample. Uncoupled Nanocage is shown in black, with the other particles coloured as in the table inset. **d**, ELISA for post-boost serum IgG as the AUC of serial dilution, from mice immunized with Homotypic SARS2 Nanocages (dark blue), Mosaic-4 (green), Mosaic-8 (light blue), SpyTag-Quartet Nanocage (pink), Dual Quartet Nanocage (purple) or Uncoupled Quartet (grey). Squares indicate ELISA against a component of that vaccine (matched) while crosses indicate ELISA against an antigen absent in that vaccine (mismatched). Responses are shown to the set of sarbecovirus RBDs, with SpyTag-MBP as a negative control. The mean is shown ±1 s.d.; *n* = 6. Individual data points and statistics are shown in Supplementary Figs. 10 and 11. Source data

We next compared the multiviral Quartet with leading mosaic nanoparticle vaccines, which have stochastic arrangements of RBDs. Mosaic-4, containing the same four RBDs as our Quartet, previously induced broad antibodies, but the best breadth was obtained with a Mosaic-8 immunogen^12,14^. Therefore, we also produced the Alternate Multiviral Quartet, containing SpyTag followed by RBDs from other sarbecoviruses: pang17, RmYN02, Rf1 and WIV1 (Supplementary Fig. 8b). Coupling both the Quartet and Alternate Quartet to SpyCatcher003-mi3 generated the Dual Quartet Nanocage, presenting the same eight RBDs as Mosaic-8 (Fig. 3a). We characterized the Alternate Quartet by SDS–PAGE/Coomassie with and without deglycosylation using PNGase F (Fig. 3b) and by size-exclusion chromatography (Supplementary Fig. 4c). To interrogate further the relationship between chain position and immunogenicity, we produced a Quartet with SpyTag moved from the C terminus to the N terminus (Supplementary Fig. 8a). This SpyTag-Quartet was used for all subsequent immunizations.

Dynamic light scattering (DLS) validated that each immunogen homogeneously assembled with SpyCatcher003-mi3 (Fig. 3c). Negative-stain transmission electron microscopy (TEM) confirmed the integrity of the Quartet Nanocages. The visible particle diameter was equivalent between uncoupled, Mosaic-8 and Quartet Nanocages, consistent with dynamic arrangement of the RBD quartet on the nanocage surface (Supplementary Fig. 9).

Immunizations compared these Mosaic and Quartet Nanocage antigens (Fig. 3a). For all RBDs, the two highest post-boost antibody titres were raised by Quartet Nanocage and Dual Quartet Nanocage (Fig. 3d and Supplementary Figs. 10–13). Surprisingly, Quartet Nanocage and Dual Quartet Nanocage induced a similar response to each other against WIV1 and pang17 (Fig. 3d and Supplementary Figs. 10–13), even though these antigens were present in Dual Quartet Nanocage but not Quartet Nanocage. In agreement with previous results^12^, Mosaic-4 and Mosaic-8 produced higher titres than SARS2 Homotypic Nanocage against the panel of sarbecovirus RBDs. Uncoupled Quartet produced similar titres as both Mosaics against the RBD set (Fig. 3d and Supplementary Figs. 10–13). These trends were also apparent in post-prime samples, except Mosaic-8 and Quartet Nanocage raised a similar anti-SARS1 response (Supplementary Fig. 10b). As previously, there was no clear relationship between chain position and antibody response against that RBD (Fig. 3d). All conditions except Uncoupled Quartet induced a comparable antibody response against SpyCatcher003-mi3 itself (Supplementary Fig. 10c). SpyTag-Maltose Binding Protein (MBP) was a negative control, revealing minimal antibodies against SpyTag itself (Supplementary Fig. 10c).

## Induction of neutralizing antibodies by Quartet Nanocages

**Fig. 4:**
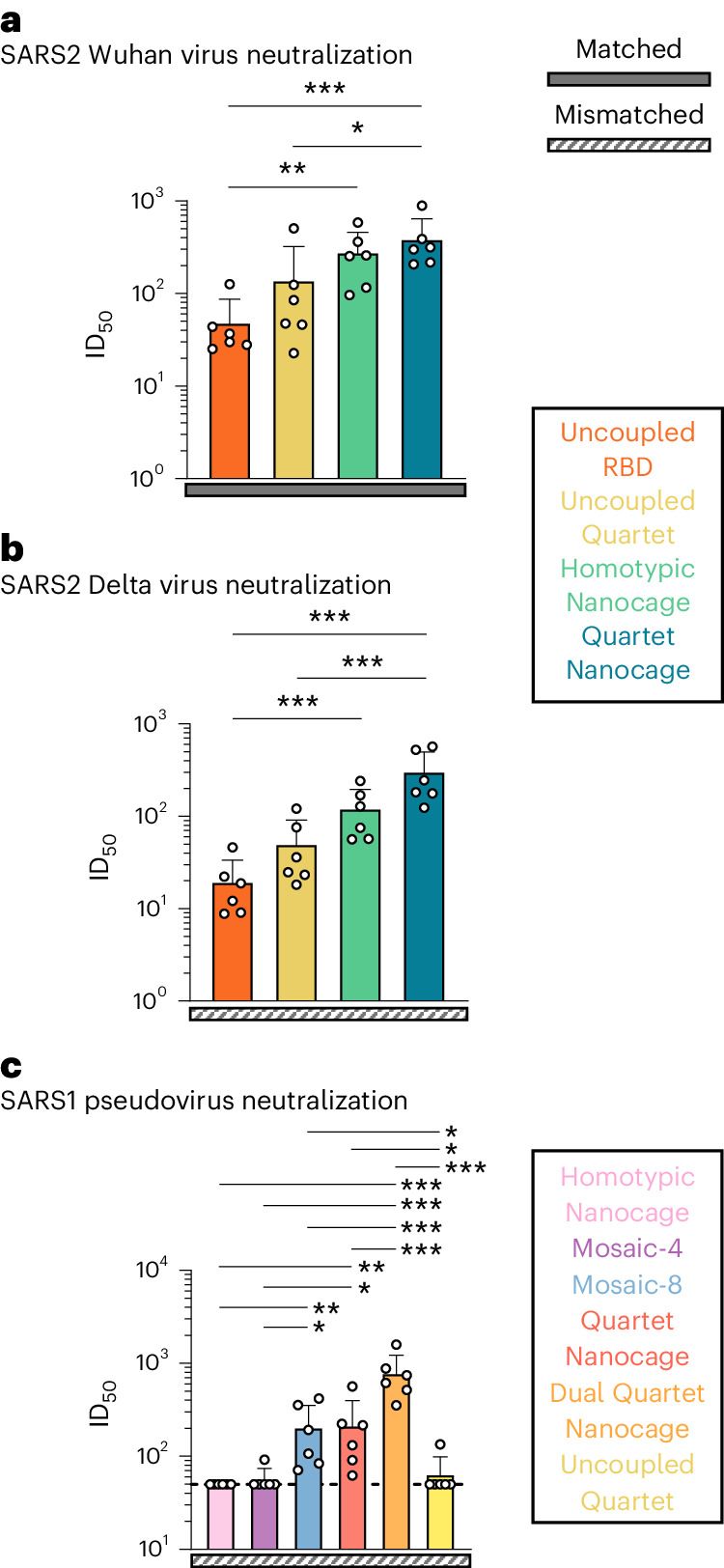
Neutralization induced by Quartet immunogens. **a**, Neutralization of Wuhan SARS2 virus by boosted mouse sera. Mice were primed and boosted with Uncoupled RBD (orange), Uncoupled Quartet (yellow), Homotypic Nanocage (green) or Quartet Nanocage (blue). Each dot represents one animal, showing the serum dilution giving 50% inhibition of infection (ID_50_). **b**, Neutralization of Delta SARS2 virus by boosted mouse sera, as in **a**. **c**, Neutralization of SARS1 pseudovirus (mismatched) by post-boost mouse sera, after immunization with different Quartet and Mosaic immunogens. Dashed horizontal lines represent the limit of detection. The mean is denoted by a bar + 1 s.d.; *n* = 6. Significance was calculated with an ANOVA test, followed by Tukey’s multiple comparison post hoc test of ID_50_ values converted to log_10_ scale. *P < 0.05, **P < 0.01, ***P < 0.001; other comparisons were non-significant. Source data

To relate antibody level to antibody efficacy, we tested neutralization of SARS2 Wuhan or Delta virus. We saw that the strongest neutralization was induced by Quartet Nanocage in each case, while Homotypic Nanocage gave higher responses than Uncoupled Quartet (Fig. 4a,b). We compared SARS1 pseudovirus neutralization induced by Quartet and Mosaic antigens, giving insight into neutralization breadth, as SARS1 was a mismatch for all tested immunogens. Pseudotyped virus neutralization correlates well with neutralization of authentic virus^20^. Dual Quartet Nanocage gave the strongest neutralizing response to SARS1. This was followed Quartet Nanocage and Mosaic-8 which induced similar, relatively strong anti-SARS1 responses (Fig. 4c).

We performed immunizations with tenfold higher antigen dose and the squalene-based adjuvant AddaVax to further enhance neutralization (Supplementary Figs. 14–17). These results are outlined further in the Supplementary Discussion.

While the motivation of this approach is protection against future zoonotic pathogens, the ideal vaccine candidate would also protect against circulating SARS2 variants. We produced an updated Kraken Quartet containing SARS2 XBB.1.5 in place of SARS2 Wuhan (Supplementary Fig. 8d). Mouse immunizations were performed with Homotypic Nanoparticles, Mosaic-8 nanoparticles and Quartet Nanocages that contained either SARS2 Wuhan or XBB.1.5, in addition to a Dual Quartet Nanocage that contained only the Wuhan RBD (Supplementary Fig. 18). All the Quartet and Mosaic immunogens produced greater antibody binding against zoonotic coronaviruses than their homotypic counterparts (Supplementary Fig. 18). There was substantial antibody binding against SARS2 VOCs with no statistically significant difference between antibody binding raised by Quartet and Mosaic immunogens against any tested SARS2 VOC (Wuhan, Delta, XBB.1.5 and BQ.1.1) (Supplementary Fig. 18). Immunogens containing XBB.1.5 provided substantially improved neutralization of SARS2 XBB.1.5 pseudovirus relative to Wuhan-containing counterparts (Supplementary Fig. 19). This result highlights the capacity to update the Quartet Nanocage, to protect against recently evolved antibody-escape variants. Both the Kraken- and Wuhan-containing Quartet Nanocage and Mosaic-8 provided greater neutralization of the mismatched SARS1 pseudovirus than their homotypic counterparts (Supplementary Fig. 19).

## Quartet Nanocage immunization in mice with existing immunity

**Fig. 5:**
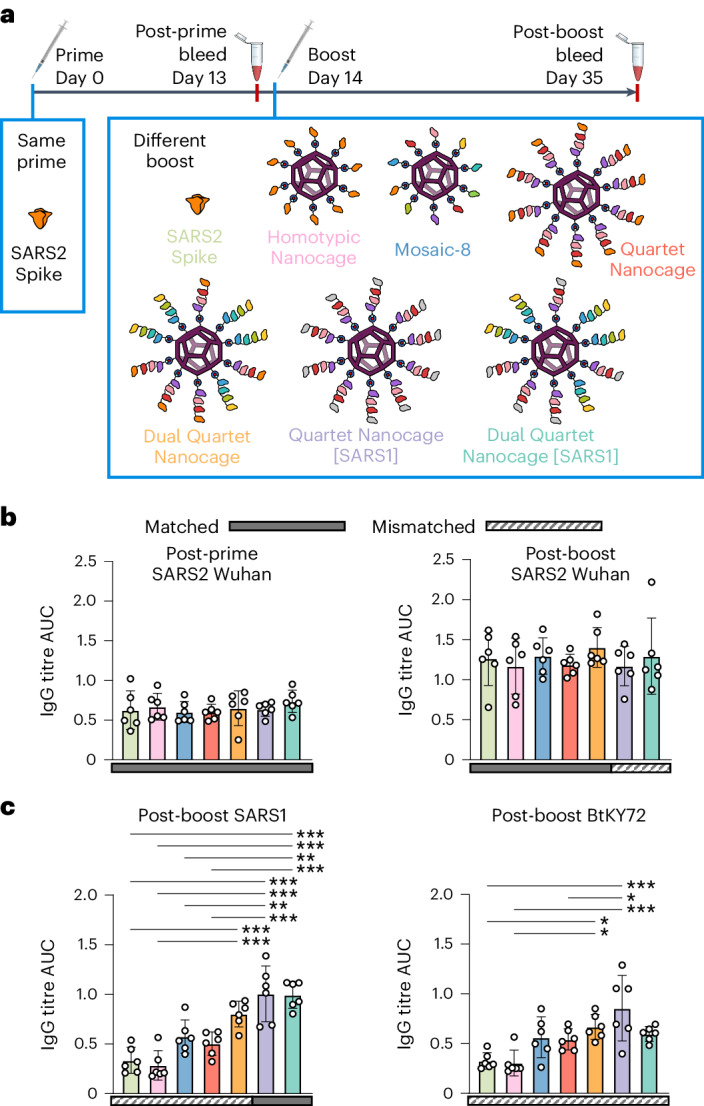
Quartet immunization induces broad antibodies even after a preprimed SARS2 response. **a**, Summary of timeline and antigens for this set of immunizations. **b**, ELISA for serum IgG to SARS2 Wuhan RBD presented as the AUC of a serial dilution. All mice were primed with Wuhan SARS2 Spike, before boosting with Wuhan SARS2 Spike protein (light green), Homotypic Nanocage (pink), Mosaic-8 (dark blue), SpyTag-Quartet Nanocage (red), Dual Quartet Nanocage (orange), Quartet Nanocage with SARS1 RBD replacing SARS2 (purple) or Dual Quartet Nanocage with SARS1 RBD replacing SARS2 (cyan). Solid rectangles under samples indicate ELISA against a component of that vaccine (matched). Striped rectangles indicate ELISA against an antigen absent in that vaccine (mismatched). Each dot represents one animal. The mean is denoted by a bar ±1 s.d.; *n* = 6. **c**, ELISA for serum IgG to other sarbecovirus RBDs, as for **b**, with each dot representing one animal and the mean being denoted by a bar ±1 s.d.; *n* = 6. Significance was calculated with an ANOVA test using Tukey’s post hoc test. *P < 0.05, **P < 0.01, ***P < 0.001; other comparisons were non-significant. Source data

Given the large fraction of the world vaccinated or previously infected with SARS2 (more than 770 million confirmed cases and 13 billion vaccine doses administered by December 2023)^21–24^, an outstanding question was whether a broad antibody response could be achieved in the face of a prebiased immune response. It is not feasible to match the pattern of vaccine sources and timings for different people around the world, but we generated a pre-existing response by priming with SARS2 Wuhan Spike (HexaPro) protein. We then boosted with different immunogens designed to elicit a broad response (Fig. 5a). One hypothesis is that animals with a pre-existing response to SARS2, upon boosting with Quartet Nanocage, would amplify their SARS2 antibodies from a memory response and be less stimulated by other antigens, so the immune response would be narrow. To test this question, we generated Quartet [SARS1], replacing SARS2 with SARS1 RBD (Supplementary Fig. 8c). This approach led to the ambitious aim of boosting a SARS2 response using an immunogen lacking any SARS2 sequence. We produced Dual Quartet Nanocage [SARS1] by mixing Alternate Quartet and Quartet [SARS1] (Supplementary Fig. 8).

Priming with SARS2 Wuhan Spike raised the expected narrow strain-specific response against SARS2 Wuhan RBD (Fig. 5b) and negligible response to SARS1 or BtKY72 (Supplementary Fig. 20). Surprisingly, the different boosts (Fig. 5b) raised similar responses against SARS2, despite SARS2 RBD being absent in Quartet Nanocage [SARS1] and Dual Quartet Nanocage [SARS1] (Fig. 5b). As expected, Quartet Nanocage [SARS1] and Dual Quartet Nanocage [SARS1] raised the strongest response against SARS1 RBD (Fig. 5c). Quartet Nanocage and Mosaic-8 raised greater antibody response than Homotypic Nanocage or Spike boost against SARS1 and BtKY72 (Fig. 5c). Mismatched responses to SARS1 and BtKY72 raised by Mosaic-8 and Quartet Nanocage were similar to the SARS1 response from a single dose of these candidates in naive mice (Supplementary Fig. 10b). Overall, Quartet Nanocages achieve broad anti-sarbecovirus response, despite animals being prebiased to a specific viral antigen. In addition, Quartet Nanocage lacking SARS2 still induces a good level of anti-SARS2 antibodies, while stimulating broad responses across sarbecoviruses.

## Further characterization of Quartet Nanocage immunogens

To investigate responses to RBDs at different distances from SpyCatcher003-mi3, we performed ELISAs on Quartet antigens using a panel of anti-SARS2 monoclonal antibodies^25^. We found minimal difference between binding to SpyTag-Quartet with or without coupling to SpyCatcher003-mi3 (Supplementary Fig. 21a,b). There was consistent reduction in anti-SARS2 antibody binding when SARS2 was the innermost RBD (Quartet-SpyTag) compared with being the outermost RBD (SpyTag-Quartet) (Supplementary Fig. 21c). Despite this difference in antibody binding, there remains no relationship between location on the chain and antibody response elicited by immunization in any condition that we tested.

Our hypothesis is that the flexible linkers facilitate a dynamic surface that displays multiple different RBDs. To this end, we produced a Quartet without flexible Gly-Ser linkers separating the different RBDs and performed immunizations comparing the No Linker Quartet Nanocage with the conventional Quartet Nanocage (Supplementary Fig. 22a). There was no statistically significant difference in the immune responses raised by the No Linker Quartet Nanocage and the conventional Quartet Nanocage to any antigen tested (Supplementary Fig. 22b). There remained no apparent relationship between location on the chain and antibody response for the No Linker Quartet Nanocage (Supplementary Fig. 22b). It remains possible that the flexible region at the N and C termini of the RBD protein maintained overall flexibility for the polyprotein.

We employed yeast-display deep mutational scanning to map mutations in SARS2 Wuhan RBD that escape antisera binding, giving insight where elicited antibodies bind (Supplementary Fig. 23)^26,27^. Homotypic Nanocage immunization produces a response dominated by narrowly focussed class 1 and class 2 antibodies^25^. The Quartet Nanocage showed variable responses: in one case class 1 dominated and other cases were dominated by class 3 or class 4 (ref. ^25^). Both the Dual Quartet Nanocage and Mosaic-8 elicited antisera binding to the evolutionarily conserved class 3 and class 4 regions (Supplementary Fig. 23).

## Conclusions

We have established that RBDs from multiple viruses can be efficiently expressed as a concatenated construct for assembly onto nanocages. These Quartet Nanocages elicited neutralizing antibodies to viruses represented on the nanocage, as well as related antigens absent from the particles. Sequential antigen repeats have mostly been explored for strings of T cell epitopes, where there is no folding to a three-dimensional structure or induction of conformation-sensitive antibodies^28^. Repeats of related structured domains may challenge the cell’s secretion machinery through undesired pairings between domains during folding^29^. However, the cell expression system here efficiently produced the various Quartets, which may be facilitated by the favourable solubility and thermostability of sarbecovirus RBDs^19^, flexibility at the domain termini and sequence divergence between the RBDs. Fusion of two SARS2 RBDs into a tandem homodimer was previously employed to enhance the immune response^30^. A tandem heterotrimer of one RBD from Wuhan, Beta and Kappa SARS2 has entered clinical development^31^. Another strategy involves fusion of individual RBDs to proliferating cell nuclear antigen to make a ring with six protruding antigens^32^. Our comparison of different chains and nanostructures indicates that tandemly linking antigens is helpful, but highly multivalent self-assembly is required for the strongest response.

We were surprised to discover no apparent relationship between RBD chain location and antibody response, despite monoclonal antibody ELISAs demonstrating differences in accessibility. Crystallography or cryoelectron microscopy do not allow clear visualization of nanostructures bearing multiple flexible regions such as the Quartet Nanocage^33^. Even SpyCatcher003-mi3 coupled to a single SpyTag-RBD showed minimal electron density for RBD in our single-particle cryoelectron microscopy^34^. The mobile termini between each RBD may provide sufficient flexibility for RBDs near to the nanocage surface to be recognized by interacting B cells. Upon immunization with Quartet Nanocage, cells with BCRs that recognize only a single type of RBD may be less likely to activate efficiently. On the other hand, BCRs recognizing features conserved in the four different RBDs on the Quartet may receive a more strongly activating stimulus. Structures have now demonstrated the molecular basis of antibody cross-recognition of diverse sarbecoviruses^35–46^.

The Mosaic-8 design was predicated on the idea that a heterogeneous arrangement of different RBDs on the nanoparticle is ideal for expansion of cross-reactive B cells. However, Mosaic-8 may face challenges in production and regulatory validation. Here, the flexibility of the Quartets may achieve a non-uniform surface for B cell stimulation while employing a uniformly made immunogen. This arrangement also presents a greater number of RBDs per nanoparticle, which may enhance antibody induction. The vaccine candidates here employ only two (Quartet Nanocage) or three (Dual Quartet Nanocage) components. Despite this, the levels and breadth of antibodies were at least comparable to, and in many cases higher than, the nine component Mosaic-8.

Omicron variants are highly effective at evading neutralizing antibodies based on natural or vaccine-derived responses to Wuhan RBD^47^, which we found after immunization with conventional Quartet Nanocage or Mosaic-8. However, Omicron XBB.1.5 RBD on a Quartet Nanocage elicited high levels of neutralizing antibodies to XBB.1.5 pseudovirus, combined with a broad anti-sarbecovirus response. This Quartet Nanocage with XBB.1.5 induced a low level of neutralization of the ancestral SARS2 D614G, illustrating the challenge in generating broad neutralization across SARS2 diversity. One route to achieve neutralization of both Omicron and ancestral strains could be to include multiple different SARS2 RBDs within a quartet. However, maintaining protection against the ancestral strain may not be a relevant vaccine limitation, since ancestral strains have been largely superseded by Omicron variants^48^.

For many diseases, notably malaria and influenza, vaccines face the challenge of inducing novel protective immunity in people with pre-existing immune responses^49,50^. After priming with SARS2 Wuhan Spike, we found that Quartet Nanocages induced an equivalent level of antibodies against Wuhan RBD as SARS2-specific immunogens. However, Quartet Nanocages additionally broadened response against diverse sarbecovirus RBDs. These data support that a Quartet Nanocage boost could be effective in a human population with existing focused immunity to SARS2.

We detected antibody induction against the SpyCatcher003-mi3 platform, which was uniform between the Homotypic, Mosaic and Quartet Nanocages. However, in contrast to viral vectored vaccines which must infect cells for their activity, for VLPs anti-platform antibodies do not impair responses against the target antigen^51,52^. VLP vaccines have generally shown a good safety margin and scalability for cost-effective global production^2,6^. Nonetheless, in future it may be valuable to apply RBD Quartets using viral vectors^53^ or messenger RNA vaccines^54^ and in pathogens beyond sarbecoviruses. Limitations of this study are that we immunized only in mice and there are differences in the vaccine candidates here compared with Mosaic-8b entering clinical trials: here, antigens were present on the nanocage at subsaturating levels with SARS2 Wuhan instead of SARS2 Beta RBD.

SARS2 has had a devastating medical and societal impact, despite the rapid generation of effective vaccines. Therefore, it is important that we possess further improved platforms for vaccination before the next major viral outbreak^55,56^. The generation of Quartet Nanocages that elicit antibodies across a range of viruses may advance proactive vaccinology, in which broadly protective vaccines are validated before the pandemic danger emerges^57^.

## Methods

### Plasmids and cloning

Cloning was performed using standard PCR methods with Q5 High-Fidelity 2× Master Mix (New England Biolabs) and Gibson assembly. All open-reading frames were validated by Sanger sequencing (Source Bioscience).

pET28a-SpyCatcher003-mi3 (GenBank MT945417, Addgene 159995) was previously described^58^. pET28a-SpyTag-MBP (GenBank MQ038699, Addgene 35050) has been published^59^. pDEST14-SpySwitch (GenBank ON131074, Addgene plasmid ID 184225) was previously described^19^. Monomeric sarbecovirus RBD expression vectors contained a C-terminal SpyTag003 (RGVPHIVMVDAYKRYK)^16^ and His_8_-tag (ref. ^12^) in the plasmid p3BNC-RBD-His8-SpyTag003 and were previously described^19^: SARS2 (GenBank ON131086), SARS1 (GenBank ON131087), RaTG13-CoV (GenBank ON131088), SHC014-CoV (GenBank ON131089), Rs4081-CoV (GenBank ON131090), pangolin17 (pang17)-CoV (GenBank ON131091), RmYN02-CoV (GenBank ON131092), Rf1-CoV (GenBank ON131093), WIV1-CoV (GenBank ON131094), Yunnan2011 (Yun11)-CoV (GenBank ON131095), BM-4831-CoV (GenBank ON131096) and BtKY72-CoV (GenBank ON131097). The origins of the sarbecovirus RBDs are SARS1 (GenBank AAP13441.1; residues 318–510), WIV1 (GenBank KF367457; residues 307–528), SHC014 (GenBank KC881005; residues 307–524), BM-4831 (GenBank NC014470; residues 310–530), BtKY72 (GenBank KY352407; residues 309–530), pang17 (GenBank QIA48632; residues 317–539), SARS2 (GenBank NC045512; S protein residues 331–529), RaTG13 (GenBank QHR63300; S protein residues 319–541), Rs4081 (GenBank KY417143; S protein residues 310–515), RmYN02 (GSAID EPI_ISL_412977; residues 298–503) and Rf1 (GenBank DQ412042; residues 310–515). The monomeric SARS2 VOC RBDs for Supplementary Fig. 18 ELISAs were cloned into pcDNA3.1 with the influenza H7 hemagglutinin (A/HongKong/125/2017) signal peptide followed by a SpyTag followed by the RBD: Wuhan (GenBank MT945427.1, Addgene 159999), Delta (GenBank PP136028, Addgene plasmid ID 214723), BQ.1.1 (GenBank PP136030, Addgene plasmid ID 214725) and XBB.1.5 (GenBank PP136029, Addgene plasmid ID 214724). The SARS2 Wuhan Spike protein was the HexaPro variant (a gift from Jason McLellan, Addgene plasmid ID 154754) that contains six proline substitutions (F817P, A892P, A899P, A942P, K986P, V987P) which confer greater stability^60^. The SARS2 Beta variant Spike protein was cloned from HexaPro to match the B.1.351 variant (L18F, D80A, D215G, ∆242-244, R246I, K417N, E484K, N501Y, D614G, A701V) in addition to the previously outlined six proline mutations. The SARS2 Delta variant Spike protein was cloned from HexaPro to match the B.1.617.2 variant (T19R, T95I, G142D, ∆156-157, R158G, L452R, T478K, D614G, P681R, D950N) in addition to the previously outlined six proline mutations.

Quartet RBD constructs were cloned using Gibson assembly in competent *Escherichia coli* DH5α cells and began with the influenza H7 hemagglutinin (A/HongKong/125/2017) signal-peptide sequence. Each RBD was separated with an eight or nine residue Gly-Ser linker. Each linker was distinct from all others in the construct to reduce potential recombination and facilitate sequence analysis. pcDNA3.1-Quartet-SpyTag was created by cloning from the N terminus to C-terminal SHC014 RBD, Rs4081 RBD, RaTG13 RBD and SARS2 RBD with a C-terminal SpyTag into pcDNA3.1 (Fig. 1c; GenBank PP136033, Addgene plasmid ID 214726). This is the construct used for Figs. 1 and 2. For subsequent figures, pcDNA3.1-SpyTag-Quartet was cloned with a SpyTag after the signal sequence and then the same order of RBDs (SpyTag-SHC014-Rs4081-RaTG13-SARS2) (Supplementary Fig. 8; GenBank PP136031, Addgene Plasmid ID 214727). pcDNA3.1-Quartet [SARS1] was cloned with SpyTag after the signal sequence, with SARS1 in the position of SARS2 (SpyTag-SHC014-Rs4081-RaTG13-SARS1) (Supplementary Fig. 8; GenBank PP136034, Addgene plasmid ID 214729). pcDNA3.1-Alternate Quartet was cloned with SpyTag after the signal sequence, followed by pang17 RBD, RmYN02 RBD, Rf1 RBD and WIV1 RBD (Supplementary Fig. 8; GenBank PP136032, Addgene plasmid ID 214728). pcDNA3.1-SpyTag-Quartet_NoLinker was cloned with the same order of RBDs as SpyTag-Quartet (SpyTag-SHC014-Rs4081-RaTG13-SARS2) but did not have any Gly-Ser linker between RBDs (Supplementary Fig. 8; GenBank PP136036, Addgene plasmid ID 214731). pcDNA3.1-Kraken Quartet was identical to SpyTag-Quartet with the SARS2 XBB.1.5 RBD in place of SARS2 Wuhan RBD (Supplementary Fig. 8; GenBank PP136035, Addgene plasmid ID 214730).

### Bacterial expression

pET28a-SpyCatcher003-mi3 or pET28a-SpyTag-MBP was transformed into *E. coli* BL21(DE3) cells (Agilent) and grown on LB-Agar plates with 50 μg ml^−1^ kanamycin for 16 h at 37 °C. A single colony was added in 10 ml of LB medium containing 50 μg ml^−1^ kanamycin and grown for 16 h at 37 °C with shaking at 200 rpm. This starter culture was then added to 1 l of LB containing 50 μg ml^−1^ kanamycin and incubated at 37 °C and with 200 rpm shaking until optical density (OD)_600_ 0.6. Cultures were induced with 0.5 mM isopropyl β-d-1-thiogalactopyranoside. For SpyCatcher003-mi3, cells were grown at 22 °C with shaking at 200 rpm for 16 h. For SpyTag-MBP, cells were grown at 30 °C with shaking at 200 rpm for 4 h. Cultures were pelleted by centrifugation at 4,000*g*.

### Purification of SpyCatcher003-mi3

Cell pellets were resuspended in 20 ml of 20 mM Tris-HCl, 300 mM NaCl, pH 8.5, supplemented with 0.1 mg ml^−1^ lysozyme, 1 mg ml^−1^ cOmplete mini EDTA-free protease inhibitor (Roche) and 1 mM phenylmethanesulfonyl fluoride. The lysate was incubated at 4 °C for 45 min with end-over-end mixing. An Ultrasonic Processor equipped with a microtip (Cole-Parmer) was used to perform sonication on ice (four times for 60 s, 50% duty-cycle). Centrifugation at 35,000*g* for 45 min at 4 °C was used to clear cell debris. Then, 170 mg of ammonium sulfate was added per ml of lysate and incubated at 4 °C for 1 h, while mixing at 120 rpm, to precipitate the particles. The solution was centrifuged for 30 min at 30,000*g* at 4 °C. The pellet was resuspended in 10 ml of mi3 buffer (25 mM Tris-HCl, 150 mM NaCl, pH 8.0) at 4 °C and filtered sequentially through 0.45 µm and 0.22 µm syringe filters (Starlab). The filtrate was dialysed for 16 h against 1,000-fold excess mi3 buffer. The dialysed particles were centrifuged at 17,000*g* for 30 min at 4 °C and filtered through a 0.22-µm syringe filter. The purified SpyCatcher003-mi3 was loaded onto a HiPrep Sephacryl S-400 HR 16-60 column (GE Healthcare), which was equilibrated with mi3 buffer using an ÄKTA Pure 25 system (GE Healthcare). The proteins were separated at 0.1 ml min^−1^ while collecting 1 ml of elution factions. The fractions containing the purified particles were pooled and concentrated using a Vivaspin 20 100 kDa molecular weight cut-off centrifugal concentrator (GE Healthcare) and stored at −80 °C.

### Mammalian protein expression

Mammalian expression of all RBD and Spike constructs was performed in Expi293F cells (Thermo Fisher, A14635). Expi293F cells were grown under humidified conditions at 37 °C and 8% (v/v) CO_2_ in Expi293 Expression Medium (Thermo Fisher) with 50 U ml^−1^ penicillin and 50 µg ml^−1^ streptomycin. Transfections were performed using the ExpiFectamine 293 Transfection Kit (Thermo Fisher). Expi293F cells were brought to 3 × 10^6 ^cells per ml and then 1 μg of plasmid DNA per ml of culture was incubated with ExpiFectamine 293 reagent for 20 min, before being added dropwise to the Expi293F culture. After approximately 20 h, ExpiFectamine 293 Transfection Enhancers 1 and 2 were added. Cell supernatants were collected after 5 d by centrifuging for 4,000*g* at 4 °C for 5 min and were passed through a 0.45 μm filter and then a 0.22 μm filter (Starlab).

### SpySwitch purification

RBDs, Quartets and SpyTag-MBP were purified by SpySwitch^19^. Purifications were performed at 4 °C. For SpyTag-MBP, cells were lysed according to the same procedure as SpyCatcher003-mi3 and supplemented with 10× SpySwitch buffer (500 mM Tris-HCl, pH 7.5, +3 M NaCl) 10% (v/v). For mammalian proteins, 10× SpySwitch buffer was added to mammalian culture supernatant at 10% (v/v). SpySwitch resin^19^, packed in an Econo-Pac Chromatography Column (Bio-Rad), was pre-equilibrated with 2 × 10 column volumes (CV) of SpySwitch buffer (50 mM Tris-HCl, pH 7.5, +300 mM NaCl). The supernatant was incubated with SpySwitch resin for 1 h at 4 °C on an end-over-end rotator. The column was washed twice with 15 CV of SpySwitch buffer. Proteins were eluted using a weakly acidic pH switch. The protein was incubated with 1.5 CV of SpySwitch Elution Buffer (50 mM acetic acid/sodium acetate, pH 5.0, +150 mM NaCl) at 4 °C with the column capped. The cap was removed and the elution flow-through was collected into a microcentrifuge tube containing 0.3 CV of 1 M Tris-HCl pH 8.0. The microcentrifuge tube was mixed by inversion to minimize the time spent at an acidic pH. This elution step was repeated for a total of six times. Purification was assessed by SDS–PAGE with Coomassie staining. Briefly, 10 µl volumes of fractions were mixed with 2 µl of 6× SDS loading buffer (234 mM Tris-HCl pH 6.8, 24% (v/v) glycerol, 120 μM bromophenol blue, 234 mM SDS), before heating at 95 °C for 5 min in a C1000 Touch Thermal Cycler (Bio-Rad) and loading onto 12% SDS–PAGE, then staining with Coomassie. Typical yields for the RBD Quartets were 50–100 mg per litre of culture. Typical yields for RBD monomers were 80–160 mg per litre of culture, as measured by bicinchoninic acid. Elution fractions were dialysed for 16 h against 1,000-fold excess Tris-buffered saline (TBS) (50 mM Tris-HCl, 150 mM NaCl, pH 7.4 at 25 °C). Proteins were stored in aliquots at −80 °C.

### Ni-NTA purification

SARS2 Spike proteins were purified by nickel-nitrilotriacetic acid (Ni-NTA) affinity chromatography. Mammalian supernatants were supplemented with 10× Ni-NTA buffer (500 mM Tris-HCl, 3 M NaCl, pH 7.8) at 10% (v/v). Ni-NTA agarose (Qiagen) was packed in an Econo-Pac Chromatography Column (Bio-Rad) and washed with 2 × 10 CV of Ni-NTA buffer (50 mM Tris-HCl, 300 mM NaCl, pH 7.8). Mammalian supernatant was incubated in the Ni-NTA column for 1 h at 4 °C with rolling. The supernatant was allowed to flow through by gravity, before being washed with 2 × 10 CV of Ni-NTA wash buffer (10 mM imidazole in Ni-NTA buffer). Elutions were performed by incubating resin with Ni-NTA elution buffer (200 mM imidazole in Ni-NTA buffer) for 5 min, before eluting by gravity. A total of six 1-CV elutions were performed. Elution fractions were assessed by SDS–PAGE with Coomassie staining, pooled and dialysed for 16 h against 1,000-fold excess TBS.

### Size-exclusion chromatography

Quartets were loaded onto a HiPrep Sephacryl S-200 HR 16-600 column (GE Healthcare), which was equilibrated with PBS pH 7.4 and run with an ÄKTA Pure 25 system (GE Healthcare). The proteins were separated at 0.5 ml min^−1^ while collecting 1 ml of elution factions. A Gel Filtration Standard (Bio-Rad) was run over the column under the same conditions for comparison. All size-exclusion chromatography was performed at 4 °C.

### PNGase F digestion

Quartet protein (2 µg) was incubated with 1 µl of Glycoprotein Denaturing Buffer (10×) (New England Biolabs) at 100 °C for 10 min with a C1000 Touch Thermal Cycler (Bio-Rad). The denatured protein was then chilled on ice for 1 min and centrifuged for 10 s at 2,000*g* with a MiniStar Silverline (VWR). Then 2 µl of GlycoBuffer 2 (10×) (New England Biolabs), 2 µl of 10% (v/v) NP-40, 6 µl of MilliQ water and 1 µl of PNGase F (New England Biolabs) at 500,000 units per ml were added and incubated at 37 °C for 1 h. Proteins were resolved on 12% SDS–PAGE, stained with Coomassie and imaged using a ChemiDoc XRS imager.

### DLS

First, 2 µM SpyTag antigens were conjugated with 2 µM SpyCatcher003-mi3 for 48 h at 4 °C. Proteins were centrifuged for 30 min at 16,900*g* at 4 °C and 30 µl of the supernatant was loaded into a quartz cuvette. Samples were measured at 20 °C using a Viscotek 802 (Viscotek) with 20 scans of 10 s each, using 50% laser intensity, 15% maximum baseline drift and 20% spike tolerance. Before collecting data, the cuvette was incubated in the instrument for 5 min to allow the sample temperature to stabilize. The intensity of the size distribution was normalized to the peak value using OmniSIZE v.3.0 software, calculating the mean and standard deviation from the multiple scans (Viscotek).

### Negative-stain TEM

First, 2 μM SpyCatcher003-mi3 was incubated for 48 h at 4 °C with 2 μM of the appropriate antigens to make Homotypic SARS2 Nanocage, Mosaic-8 and Quartet Nanocage or without any antigen present in 25 mM Tris-HCl, 150 mM NaCl, pH 8.0. Samples were applied to a freshly glow-discharged TEM grid, blotted twice with water and stained with 2% (w/v) uranyl acetate for 30 s. Samples were imaged using a Tecnai G2 80–200-keV transmission electron microscope at the Cambridge Advanced Imaging Centre. For size analysis, the particle diameter for each group was measured manually (*n* = 75) and plotted with 2-nm bin size in Excel (Microsoft).

### Endotoxin depletion and quantification

Endotoxin was removed from all vaccine components using Triton X-114 phase separation^61,62^. Triton X-114 at a final 1% (v/v) was added to the protein on ice and incubated for 5 min. The solution was incubated at 37 °C for 5 min and centrifuged for 1 min at 16,000*g* at 37 °C. The top phase was transferred to a fresh tube. This procedure was repeated for a total of three times. A final repetition without the addition of Triton X-114 was performed, to account for residual Triton X-114. A Pierce Chromogenic Endotoxin Quant Kit (Thermo Fisher) was used according to manufacturer instructions to quantify the final endotoxin concentration. All vaccine components were below the accepted endotoxin levels for vaccine products of 20 endotoxin units per ml (ref. ^63^).

### Immunogen preparation

The concentration of vaccine components was measured using bicinchoninic acid assay (Pierce). Where multiple antigens were coupled to the nanocage, the antigens were first mixed in equimolar amounts in TBS. Doses were normalized by the number of SpyTags, to facilitate an equimolar amount of SpyCatcher003-mi3 nanocages with similar occupancy in each condition. For high-dose immunizations (Supplementary Figs. 14–16), SpyCatcher003-mi3 at 8 µM was incubated with 8 µM SpyTagged antigen for 48 h at 4 °C in TBS, pH 8.0. For other immunizations, SpyCatcher003-mi3 at 0.8 µM was incubated with 0.8 µM total SpyTagged antigen for 48 h at 4 °C in TBS, pH 8.0. Uncoupled RBD and Uncoupled Quartet were incubated at 0.8 µM for 48 h at 4 °C in TBS, pH 8.0, without the addition of SpyCatcher003-mi3. Before immunization, samples were analysed by SDS–PAGE/Coomassie and DLS. For Fig. 5, SARS2 Spike prime and boost doses were performed with 10 µg of SARS2 Wuhan Spike (HexaPro) protein in TBS pH 8.0 at 4 °C.

### Mouse immunization and blood sampling

Animal experiments were performed according to the UK Animals (Scientific Procedures) Act 1986, under Project License (PBA43A2E4 and PP9362617) and approved by the University of Oxford Animal Welfare and Ethical Review Body. Mice that were 6 weeks old (at the time of the first immunization) were obtained from Envigo. For high-dose immunizations (Supplementary Figs. 14–16), we used BALB/c female mice, and for all other immunizations we used C57BL/6 female mice. Mice were housed in accordance with the UK Home Office ethical and welfare guidelines and fed on standard chow and water ad libitum. Before immunization, immunogens were mixed 1:1 with VAC 20 adjuvant (SPI Pharma) (25 µl + 25 µl), except for the high-dose immunizations (Supplementary Figs. 14–16) where immunogens were mixed 1:1 with AddaVax (Invivogen). This procedure gave final doses of 0.2 nmol of total SpyTagged antigen for high-dose immunizations and 0.02 nmol of total SpyTagged antigen for normal-dose immunization. For normal-dose immunization, this relates to 0.6 µg of Uncoupled RBD. Isoflurane (Abbott)-anaesthetised mice were immunized on day 0 and day 14 intramuscularly in the gastrocnemius muscle with the specified antigen–adjuvant mix. Post-prime blood samples were obtained on day 13 via tail vein using Microvette (CB300, Sarstedt) capillary tubes. Post-boost samples were obtained on days 32 to 41 (exact day for each set of immunizations is indicated in the figure) via cardiac puncture of humanely killed mice. The collected whole blood in microtainer SST tubes (Becton Dickinson) was allowed to clot at 25 °C for 1–2 h, before spinning down at 10,000*g* for 5 min at 25 °C. The sera were heat-inactivated at 56 °C for 30 min, before storing at −20 °C.

### Mouse antisera ELISA

Nunc MaxiSorp plates (Thermo Fisher) were coated with 80 nM purified SpyTag-RBD, SpyTag-MBP or SpyCatcher003-mi3 in PBS (137 mM NaCl, 2.7 mM KCl, 10 mM Na_2_HPO_4_, 1.7 mM KH_2_PO_4_, pH 7.4) at 4 °C for 16 h. Where SARS2 was analysed, this refers to the Wuhan variant, unless indicated. In Supplementary Fig. 5b, the response to different SARS2 variants was measured by coating 1 µg ml^−1^ of the indicated HexaPro Spike protein in PBS and incubating at 4 °C for 16 h. Plates were washed three times with PBS supplemented with 1% (v/v) Tween 20 (PBST). Plates were blocked by 2 h of incubation at 25 °C with 5% (w/v) skimmed milk in PBS. Plates were then washed three times with PBST. Sera were serially diluted into the blocking buffer using eight-point, fourfold series starting at 1:100. Plates were incubated with sera for 1 h at 25 °C and then washed three times with PBST. Plates were incubated at 25 °C for 1 h with a 1:1,600 dilution of horseradish peroxidase-conjugated goat anti-mouse IgG antibody (Sigma-Aldrich, A9044). Plates were washed three times with PBST. Plates were then incubated at 25 °C for 5 min with 1-Step Ultra TMB-ELISA Substrate Solution (Thermo Scientific) before the reaction was stopped with 1 M H_2_SO_4_. Absorbance measurements at 405 nm (*A*_405_) were taken with a FLUOstar Omega plate reader (BMG Labtech) using Omega MARS software (BMG Labtech). A sigmoidal dose–response curve was fit to the absorbance data using the optimize.curve_fit() function from the Python SciPy library^64^. The sigmoidal dose–response function was:

$${{y}}={\rm{Bottom}}+\frac{{\rm{Top}}-{\rm{Bottom}}}{1+{10}^{{\log }_{10}\left({{\rm{IC}}}_{50}\right)-{{x}}}}$$

IC_50_ is the serum concentration that gives a 50% signal between the maximum and minimum of the curve. The area under the fitted curve was determined using the trapz function from the Python NumPy library^65^. Area under the curve was used instead of endpoint titre to account better for data across the entire range of values^66^. For calculation of midpoint titre, sigmoidal dose–response curve absorbance data and the midpoint were calculated using GraphPad Prism (GraphPad Software v.9.4.1). Results were plotted using GraphPad Prism (GraphPad Software v.9.4.1).

### Microneutralization assay

These assays were performed in the James & Lillian Martin Centre, University of Oxford, operating under license from the Health and Safety Authority, UK, on the basis of an agreed Code of Practice, Risk Assessments (under the Advisory Committee on Dangerous Pathogens) and standard operating procedures. The microneutralization assay determines the serum concentration that induces a 50% reduction in focus-forming units of SARS2 in Vero cells (American Type Culture Collection, CCL-81). A serial dilution of immunization sera (seven steps from 1/40 to 1/40,000 diluted into DMEM) was pre-incubated for 30 min at 25 °C with a fixed dose of 100–200 focus-forming units (20 μl) of different authentic SARS2 variants. This procedure was performed in triplicate for samples from high-dose immunizations outlined in Supplementary Figs. 14–16 and in quadruplicate for all other samples. DMEM on its own was used for serum-free control wells, which were used to define 100% infectivity. The Victoria 01/2020 isolate (Pango B) was used for Wuhan neutralization^67^. The Beta variant (Pango B.1.351) used for neutralizations is the HV001 isolate, sequenced and kindly supplied by CAPRISA, Durban, South Africa^68^. The isolates for Delta (Pango B.1.617.2), Omicron BA.1 (Pango B.1.1.529.1) and Omicron BQ.1.1 (Pango B.1.1.529.5.3.1.1.1.1.1.1) were kindly supplied by Gavin Screaton (University of Oxford). This mixture was incubated with 100 μl of Vero cells (4.5 × 10^4^) at 37 °C with 5% (v/v) CO_2_. At 2 h into this incubation, a 1.5% (w/v) carboxymethyl cellulose-containing overlay was applied to prevent satellite focus formation. At 18 h post-infection, the monolayers were fixed with 4% (w/v) paraformaldehyde in PBS and then permeabilized with 2% (v/v) Triton X-100. The cells were stained using the FB9B monoclonal antibody at 1 µg ml^−1^ (ref. ^69^). These samples were developed using an anti-human IgG (Fc-specific) peroxidase-conjugated antibody (1:5,000 dilution, cat. no. A0170-1ML, Sigma-Aldrich) and True Blue peroxidase substrate. The infectious foci were enumerated by Classic ELISpot Reader (AID GmbH). Data were analysed using four-parameter logistic regression (Hill equation) using GraphPad Prism (GraphPad Software v.8.3). Statistical significance of differences between groups was determined using a one-way analysis of variance (ANOVA) test, followed by Tukey’s multiple comparison post hoc test of half-maximal inhibitory dilution (ID_50_) values converted to log_10_ scale using GraphPad Prism (GraphPad Software v.9.4.1).

### Pseudovirus neutralization assay

SARS2 BQ.1.1, SARS1, WIV1, SHC014 and BtKY72 K493Y/T498W pseudotyped viruses were prepared as described^70,71^. The double mutation BtKY72 K493Y/T498W in the BtKY72 Spike protein has previously been shown to enable entry to human cells via ACE2 (ref. ^72^). This technique for producing pseudoviruses employs HIV-based lentiviral particles with genes encoding the appropriate Spike protein lacking the cytoplasmic tail. A threefold serial dilution of sera was incubated with pseudotyped virus for 1 h at 37 °C. The mixture was incubated with 293T_ACE2_ target cells for 48 h at 37 °C (ref. ^12^). Cells were washed twice with PBS, before being lysed with Luciferase Cell Culture Lysis 5× reagent (Promega). NanoLuc Luciferase activity in the lysates was measured using the Nano-Glo Luciferase Assay System (Promega). The relative luminescence units were normalized to values derived from cells infected with pseudotyped virus in the absence of serum. ID_50_ was determined using four-parameter nonlinear regression in AntibodyDatabase^73^ and plotted using GraphPad Prism (GraphPad Software v.9.4.1). Statistical significance of differences between groups was determined using an ANOVA test, followed by Tukey’s multiple comparison post hoc test of ID_50_ values converted to log_10_ scale using GraphPad Prism (GraphPad Software v.9.4.1).

### Monoclonal antibody ELISAs

We incubated 2 μM SpyTag-Quartet or 2 μM Quartet-SpyTag with or without 2 μM SpyCatcher003-mi3 in 25 mM Tris-HCl, 150 mM NaCl, pH 8.0, for 16 h at 4 °C to allow for coupling. We added the protein samples at 50 nM to Nunc MaxiSorp plates (Thermo Fisher) and incubated for 16 h at 4 °C in PBS pH 7.4. We then washed three times with PBST and blocked with 5% (w/v) skim milk for 2 h at 25 °C. We washed three times more and incubated with 50 nM of the specified antibody for 1 h at 25 °C. The monoclonal antibodies used in this study, namely C121 71, EY-6A, FI-3A, FP-12A, IY-2A (ref. ^45^), LCA60 (ref. ^74^), FP-8A and FD-5D (refs. ^19,69^), have all been previously described. Heavy and light chain expression vectors for these antibodies were co-transfected into ExpiCHO cells (Thermo Fisher Scientific, A29133) using the ExpiCHO expression system kit, and the monoclonal antibodies were purified from the supernatant by Protein A Sepharose (GE Healthcare). After three washes, we incubated with a 1/2,500 dilution of anti-human IgG horseradish peroxidase (Sigma-Aldrich, A8667) for 1 h at 25 °C. After three washes, we incubated with TMB for 30 s (for comparison of coupled and uncoupled Quartet) or 2 min (for comparison of SpyTag-Quartet and Quartet-SpyTag), before stopping with 1 M HCl. *A*_405_ measurements of triplicate wells per condition were taken at 25 °C with a FLUOstar Omega plate reader (BMG Labtech) using Omega MARS software (BMG Labtech).

### Deep mutational scanning

Serum mapping studies were performed following the previously established approach^26^: 25 µl of each serum sample was heat-inactivated for 30 min at 56 °C, before depleting twice by incubation with 50 OD units of AWY101 yeast containing an empty vector, to deplete serum of non-specific yeast-binding antibodies. Yeasts that were generously provided by Tyler Starr (University of Utah) were induced to express the SARS2 RBD library in galactose-containing synthetic defined medium with casamino acids: 6.7 g l^−1^ Yeast Nitrogen Base, 5.0 g l^−1^ casamino acids, 1.065 g l^−1^ 2-(*N*-morpholino)ethanesulfonic acid (MES), 2% (w/v) galactose and 0.1% (w/v) dextrose^26^. After a 16–18-h induction, cells were washed and incubated with serum at a range of dilutions for 1 h at 25 °C with gentle agitation. For each serum sample, a subsaturating dilution enabled the fluorescent signal from antibody binding to be equivalent across samples. The libraries were washed and labelled for 1 h with 1:100 fluorescein-conjugated anti-myc tag antibody (Immunology Consultants Lab, CYMC-45F) to quantify RBD expression and 1:200 Alexa Fluor-647-goat anti-mouse-IgG Fc-gamma (Jackson ImmunoResearch, 115-605-008) to detect mouse antibodies from serum. Approximately 5 × 10^6^ RBD-positive cells were processed on a Sony SH800 cell sorter. A flow cytometric gate was drawn to capture RBD mutants with reduced antibody binding compared with their level of RBD expression^26^. These cells were grown overnight, before plasmid extraction in a synthetic defined medium with casamino acids: 6.7 g l^−1^ Yeast Nitrogen Base, 5.0 g l^−1^ casamino acids, 1.065 g l^−1^ MES, 2% (w/v) dextrose, 100 U ml^−1^ penicillin and 100 µg ml^−1^ streptomycin. Plasmid samples were then prepared from 30 OD units (1.6 × 10^8^ colony forming units; cfu) of preselection yeast populations and 5 OD units (~3.2 × 10^7^ cfu) of overnight cultures of serum-escaped cells (Zymoprep Yeast Plasmid Miniprep II)^26^. The 16-nucleotide barcodes identifying each RBD variant were amplified by PCR and sequenced on an Illumina HiSeq 2500 with 50-base pair single-end reads^26^. We computationally filtered out variants with >1 amino acid mutation, low sequencing counts or highly deleterious mutations that might escape antibody binding because of poor RBD expression or folding^26^. The escape fraction represents the proportion of cells expressing that specific variant that falls in the escape bin: a value of 0 means that the variant is always bound by serum antibody and a value of 1 means that the variant always escapes serum antibody binding. The height of each letter indicates the escape fraction for that amino acid mutation, calculated as described above. The static logo plots feature any site where, for at least one serum sample, the site-total antibody escape was >10× the median across all sites and at least 10% the maximum of any site. RBD sites are categorized based on antibody epitope region^28^. Class 1 epitopes are defined as residues 403, 405, 406, 417, 420, 421, 453, 455–460, 473–478, 486, 487, 489, 503 and 504. Class 2 epitopes are defined as residues 472, 479, 483–485 and 490–495. Class 3 epitopes are defined as residues 341, 345, 346, 354–357, 396, 437–452, 466–468, 496, 498–501 and 462. Class 4 epitopes are defined as residues 365–390 and 408.

### Bioinformatics

The phylogenetic tree of sarbecovirus RBD sequences was constructed using MEGA X v.11.0.13 software^75^. Multiple sequence alignment and calculation of amino acid identity were performed using Clustal Omega v.1.2.4 (ref. ^76^). The structure of SARS2 RBD was based on PDB ID: 6ZER (ref. ^77^) and analysed using PyMOL v.2.5.2.

### Statistics and reproducibility

No statistical method was used to predetermine sample size. Significance for ELISAs was calculated with an ANOVA test using Tukey’s post hoc test in GraphPad Prism (GraphPad Software v.9.4.1). Comparisons for neutralizations were calculated with an ANOVA test, followed by Tukey’s multiple comparison post hoc test of ID_50_ values converted to log_10_ scale using GraphPad Prism (GraphPad Software v.9.4.1). For ELISAs and neutralizations, Tukey’s test was used to correct for the multiple comparisons between the responses raised to each individual antigen within each set of immunizations. Significance was assigned according to: *P < 0.05, **P < 0.01, ***P < 0.001. On graphs where some conditions are compared, where no test is marked then the difference was non-significant. The experiments were not randomized. The investigators were not blinded to allocation during experiments and outcome assessment.

### Reporting summary

Further information on research design is available in the Nature Portfolio Reporting Summary linked to this article.

## Online content

Any methods, additional references, Nature Portfolio reporting summaries, source data, extended data, supplementary information, acknowledgements, peer review information; details of author contributions and competing interests; and statements of data and code availability are available at 10.1038/s41565-024-01655-9.

### Supplementary information

Supplementary Information Supplementary Figs. 1–23 and Discussion.

Reporting Summary

Supplementary Fig. 3 Unprocessed gels.

Supplementary Figs. 3–7 and 9–23 Source data and statistical data.

### Source data

Source Data Fig. 1 Unprocessed gels.

Source Data Fig. 2 Unprocessed gels.

Source Data Fig. 2 Source data and statistical data.

Source Data Fig. 3 Source data.

Source Data Fig. 4 Source data and statistical data.

Source Data Fig. 5 Source data and statistical data.

## Data Availability

Sequences of constructs are available in GenBank, as described in the section ‘Plasmids and cloning’. Plasmids encoding pDEST14-SpySwitch, pET28a-SpyCatcher003-mi3, pET28a-SpyTag-MBP, pcDNA3.1-SpyTag-SARS2 RBD Wuhan, pcDNA3.1-SpyTag-SARS2 RBD Delta, pcDNA3.1-SpyTag-SARS2 RBD BQ.1.1, pcDNA3.1-SpyTag-SARS2 RBD XBB.1.5, pcDNA3.1-SpyTag-Quartet, pcDNA3.1-Alternate Quartet, pcDNA3.1-Kraken Quartet, pcDNA3.1-Quartet [SARS1] and pcDNA3.1-SpyTag-Quartet_No_Linker have been deposited in the Addgene repository (https://www.addgene.org/Mark_Howarth/). Source data are provided with this paper. Requests for further information and/or resources and reagents should be directed to and will be fulfilled by the lead contact, M.R.H. (mh2186@cam.ac.uk).
