# Supplementary material for: Proactive vaccination using multiviral Quartet Nanocages to elicit broad anti-coronavirus responses
Source: Nat Nanotechnol. 2024 May 6;19(8):1216–23. doi: 10.1038/s41565-024-01655-9 (PMC11329374; doi:10.1038/s41565-024-01655-9)
Supplement: Supplementary file 1 — Supplementary Figs. 1–23 and Discussion. [file 41565_2024_1655_MOESM1_ESM.pdf]

nature nanotechnology

Article

https://doi.org/10.1038/s41565-024-01655-9

Proactive vaccination using multiviral Quartet Nanocages to elicit broad anti-coronavirus responses

In the format provided by the authors and unedited

```
         320                340                 360                 380                400
Rs4081   RVSPTHEVVRFPNITNRCPFDKVFNASRFPNVYAWERTKISDCVADYTVLYNS-TSFSTFKCYGVSPSKLIDLCFTSVYADTFL
RmYN02   RILPSTEVVRFPNITNFCPFDKVFNATRFPNVYAWQRTKISDCIADYTVLYNS-TSFSTFKCYGVSPSKLIDLCFTSVYADTFL
Rf1      RVSPVTEVVRFPNITNLCPFDKVFNATRFPSVYAWERTKISDCVADYTVFYNS-TSFSTFNCYGVSPSKLIDLCFTSVYADTFL
BM-4831  RVTPTTEVVRFPNITQLCPFNEVFNITSFPSVYAWERMRITNCVADYSVLYNSSASFSTFQCYGVSPTKLNDLCFSSVYADYFV
BtKY72   RVSPSTEVVRFPNITNLCPFGQVFNASNFPSVYAWERLRISDCVADYAVLYNSSSSFSTFKCYGVSPTKLNDLCFSSVYADYFV
pang17   RVQPTISIVRFPNITNLCPFGEVFNASKFASVYAWNRKRISNCVADYSVLYNS-TSFSTFKCYGVSPTKLNDLCFTNVYADSFV
SARS2    RVQPTESIVRFPNITNLCPFGEVFNATRFASVYAWNRKRISNCVADYSVLYNS-ASFSTFKCYGVSPTKLNDLCFTNVYADSFV
RaTG13   RVQPTDSIVRFPNITNLCPFGEVFNATTFASVYAWNRKRISNCVADYSVLYNS-TSFSTFKCYGVSPTKLNDLCFTNVYADSFV
SHC014   RVAPSKEVVRFPNITNLCPFGEVFNATTFPSVYAWERKRISNCVADYSVLYNS-TSFSTFKCYGVSATKLNDLCFSNVYADSFV
SARS1    RVVPSGDVVRFPNITNLCPFGEVFNATKFPSVYAWERKKISNCVADYSVLYNS-TFFSTFKCYGVSATKLNDLCFSNVYADSFV
WIV1     RVAPSKEVVRFPNITNLCPFGEVFNATTFPSVYAWERKRISNCVADYSVLYNS-TSFSTFKCYGVSATKLNDLCFSNVYADSFV
         *: *  .::******: ***..*** : * .****:* :*::*:***:*:*** : ****:***** :** ****:.**** *:

                          420                440                460                480
Rs4081   IRSSEVRQVAPGETGVIADYNYKLPDDFTGCVIAWNTAKQDQG-----QYYYRSSRKTKLKPFERDLTSDE-------------
RmYN02   IRFSEVRQIAPGETGVIADYNYKLPDDFTGCVLAWNTAQQDIG-----SYFYRSHRAVKLKPFERDLSSDE-------------
Rf1      IRFSEVRQVAPGQTGVIADYNYKLPDDFTGCVIAWNTAKQDVG-----SYFYRSHRSSKLKPFERDLSSEE-------------
BM-4831  VKGDDVRQIAPAQTGVIADYNYKLPDDFTGCVIAWNTNSLDS--SNE--FFYRRFRHGKIKPYGRDLSNVLFNPSGGTCSA-EG
BtKY72   VKGDDVRQIAPAQTGVIADYNYKLPDDFTGCVLAWNTNSVDSKSGNN--FYYRLFRHGKIKPYERDISNVLYNSAGGTCSSISQ
pang17   VKGDEVRQIAPGQTGVIADYNYKLPDDFTGCVIAWNSVKQDALTGGNYGYLYRLFRKSKLKPFERDISTEIYQAGSTPCNGQVG
SARS2    IRGDEVRQIAPGQTGKIADYNYKLPDDFTGCVIAWNSNNLDSKVGGNYNYLYRLFRKSNLKPFERDISTEIYQAGSTPCNGVEG
RaTG13   ITGDEVRQIAPGQTGKIADYNYKLPDDFTGCVIAWNSKHIDAKEGGNFNYLYRLFRKANLKPFERDISTEIYQAGSKPCNGQTG
SHC014   VKGDDVRQIAPGQTGVIADYNYKLPDDFLGCVLAWNTNSKDSSTSGNYNYLYRWVRRSKLNPYERDLSNDIYSPGGQSCSA-VG
SARS1    VKGDDVRQIAPGQTGVIADYNYKLPDDFMGCVLAWNTRNIDATSTGNYNYKYRYLRHGKLRPFERDISNVPFSPDGKPCTP-PA
WIV1     VKGDDVRQIAPGQTGVIADYNYKLPDDFTGCVLAWNTRNIDATQTGNYNYKYRSLRHGKLRPFERDISNVPFSPDGKPCTP-PA
         :  .:***:**.:** **********:* ***:***:   *        : **  *  ::.*: **::.

                       500                520                540
Rs4081    -NGVRTLSTYDFYPNVPIEYQATRVVVLSFELLNAPATVCGPKLSTALVKNQCVNF
RmYN02    -NGVRTLSTYDFNPNVPLDYQATRVVVLSFELLNAPATVCGPKLSTQLVKNRCVNF
Rf1       -NGVRTLSTYDFNQNVPLEYQATRVVVLSFELLNAPATVCGPKLSTSLVKNQCVNF
BM-4831   LNCYKPLASYGFTQSSGIGFQPYRVVVLSFELLNAPATVCGPKQSTELVKNKCVNF
BtKY72    LGCYEPLKSYGFTPTVGVGYQPYRVVVLSFELLNAPATVCGPKKSTELVKNKCVNF
pang17    LNCYYPLERYGFHPTTGVNYQPFRVVVLSFELLNGPATVCGPKLSTTLVKDKCVNF
SARS2     FNCYFPLQSYGFQPTNGVGYQPYRVVVLSFELLHAPATVCGPKKSTNLVKNKCVNF
RaTG13    LNCYYPLYRYGFYPTDGVGHQPYRVVVLSFELLNAPATVCGPKKSTNLVKNKCVNF
SHC014    PNCYNPLRPYGFFTTAGVGHQPYRVVVLSFELLNAPATVCGPKLSTDLIKNQCVNF
SARS1     LNCYWPLNDYGFYTTTGIGYQPYRVVVLSFELLNAPATVCGPKLSTDLIKNQCVNF
WIV1      FNCYWPLNDYGFYITNGIGYQPYRVVVLSFELLNAPATVCGPKLSTDLIKNQCVNF
           .    *  *.*  .  : .*  **********:.******** ** *:*::****
```

* = fully conserved
: = strongly similar
. = weakly similar

Supplementary Fig. 1. Sarbecovirus RBD sequence alignment. Amino acid sequence alignment of sarbecovirus RBDs used in this study, numbered according to Spike protein of SARS2 Wuhan variant.

A Sarbecovirus RBD Protein Identity

Clade		Rs4081	RmYN02	Rf1	BM-4831	BtKY72	pang17	RaTG13	SARS2 Beta	SARS2 Wuhan	SARS2 Delta	SARS2 BA.1	SARS2 BQ.1.1	SARS2 XBB.1	SARS2 XBB.1.5	SHC014	SARS1	WIV1
2	Rs4081	100	88	89	67	70	71	69	69	69	69	68	66	65	65	68	68	70
2	RmYN02		100	90	67	70	69	68	68	69	68	66	65	64	64	69	69	70
2	Rf1			100	70	70	71	70	70	71	70	69	68	67	67	70	70	71
3	BM-4831				100	83	70	71	71	72	71	69	68	68	68	77	74	75
3	BtKY72					100	73	73	73	74	73	70	69	69	69	77	75	76
1b	pang17						100	88	87	87	86	83	83	82	82	77	75	75
1b	RaTG13							100	89	90	90	87	86	85	85	76	75	77
1b	SARS2 Beta								100	99	98	94	92	91	91	76	73	75
1b	SARS2 Wuhan									100	99	93	91	90	90	76	73	76
1b	SARS2 Delta										100	93	91	90	90	76	74	76
1b	SARS2 BA.1											100	96	94	94	75	71	72
1b	SARS2 BQ.1.1												100	96	96	75	71	73
1b	SARS2 XBB.1													100	100	74	70	71
1b	SARS2 XBB.1.5														100	75	70	71
1a	SHC014															100	82	84
1a	SARS1																100	95
1a	WIV1																	100

RBD Amino Acid Identity: 100% / 90% / 80% / 70% / 60%

B Map of Residue Conservation

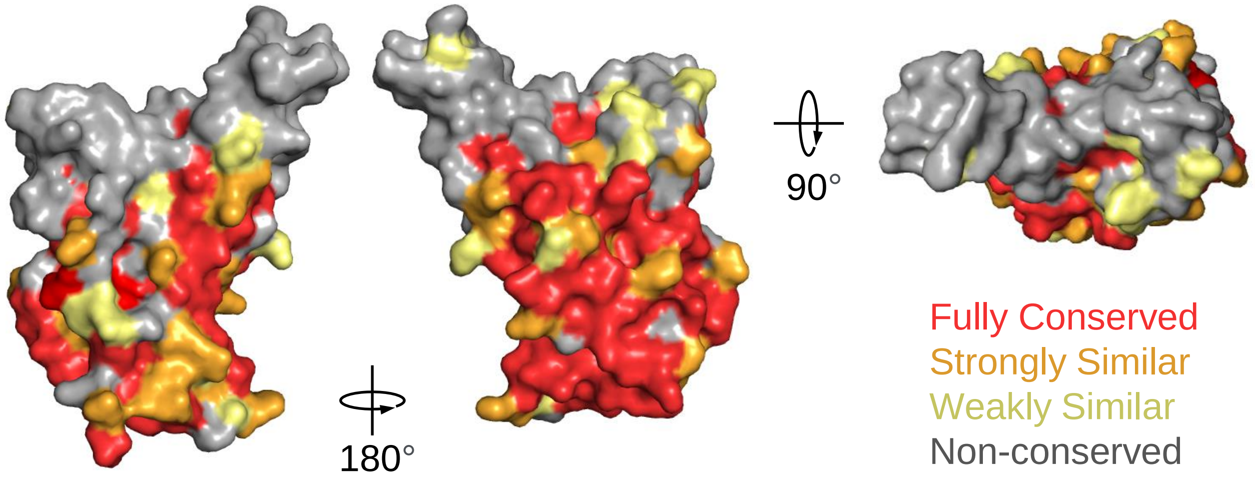

Supplementary Fig. 2. RBD residue conservation. (**A**) Heat map of percent amino acid identity between sarbecovirus RBDs used in this study. (**B**) Conservation of residues between sarbecoviruses used in the study, as mapped onto the SARS2 Wuhan RBD crystal structure (PDB ID: 6ZER). Multiple orientations of the same RBD are shown, represented as the van der Waals surface.

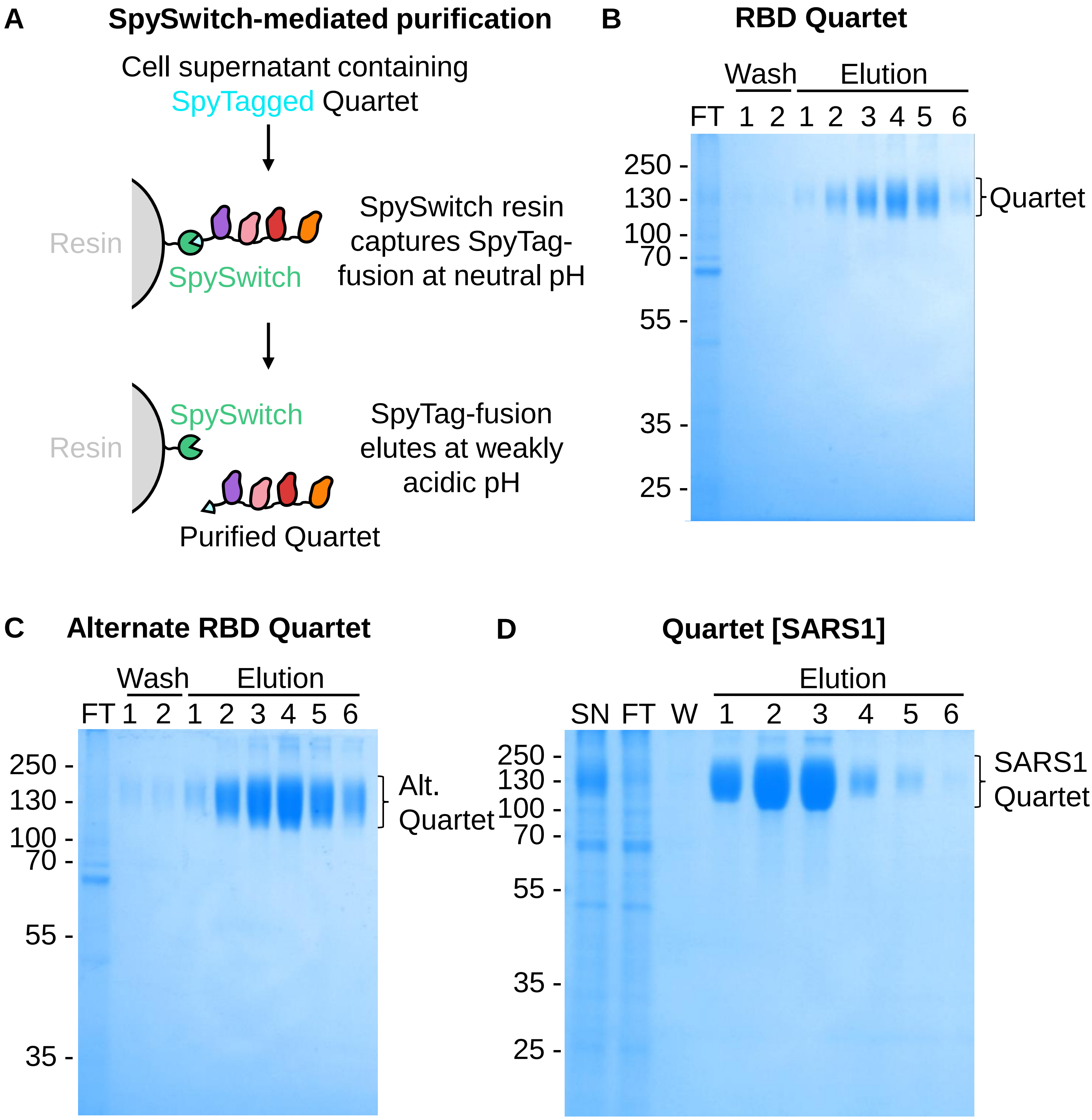

Supplementary Fig. 3. SpySwitch purification of RBD quartets. (**A**) Schematic of SpySwitch affinity purification. SpyTag genetically fused to the Quartet has a non-covalent interaction with SpySwitch at a neutral pH, before eluting at a weakly acidic pH through charge-charge repulsion. This system was used to purify (**B**) RBD Quartet, (**C**) Alternate RBD Quartet, and (**D**) RBD Quartet with SARS1 in place of SARS2. The supernatant (SN), flowthrough (FT), wash (W) and elution fractions were analyzed by SDS-PAGE with Coomassie staining. A representative gel from two independent purifications. Molecular weight markers are in kDa.

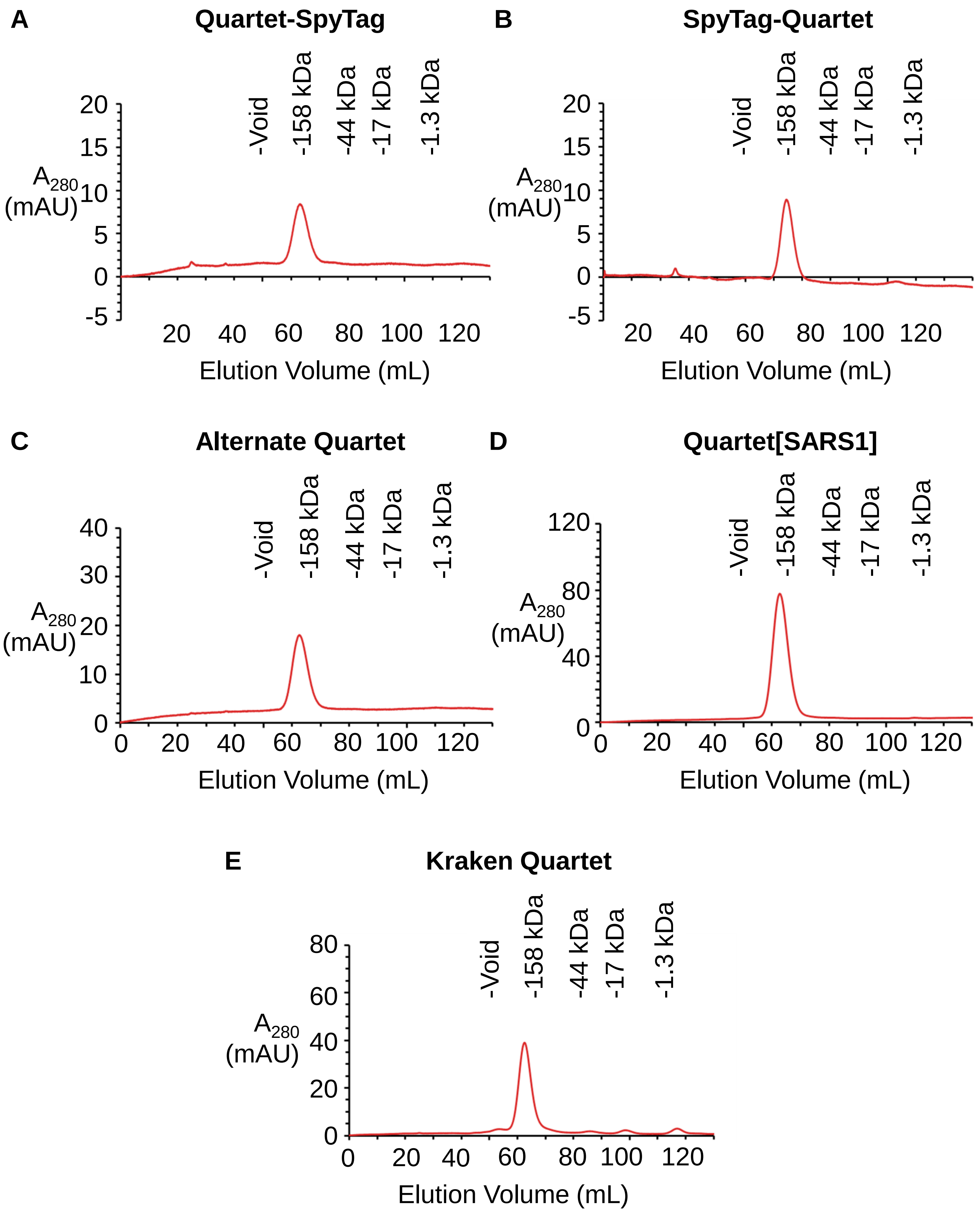

Supplementary Fig. 4. Size-exclusion chromatography of Quartets. Quartets were run on an S200 Sephacryl column in PBS pH 7.4 after purification by SpySwitch, with gel filtration standards shown. Absorbance is measured in mAU. Absorbance peak size differs in line with the different loaded concentrations.

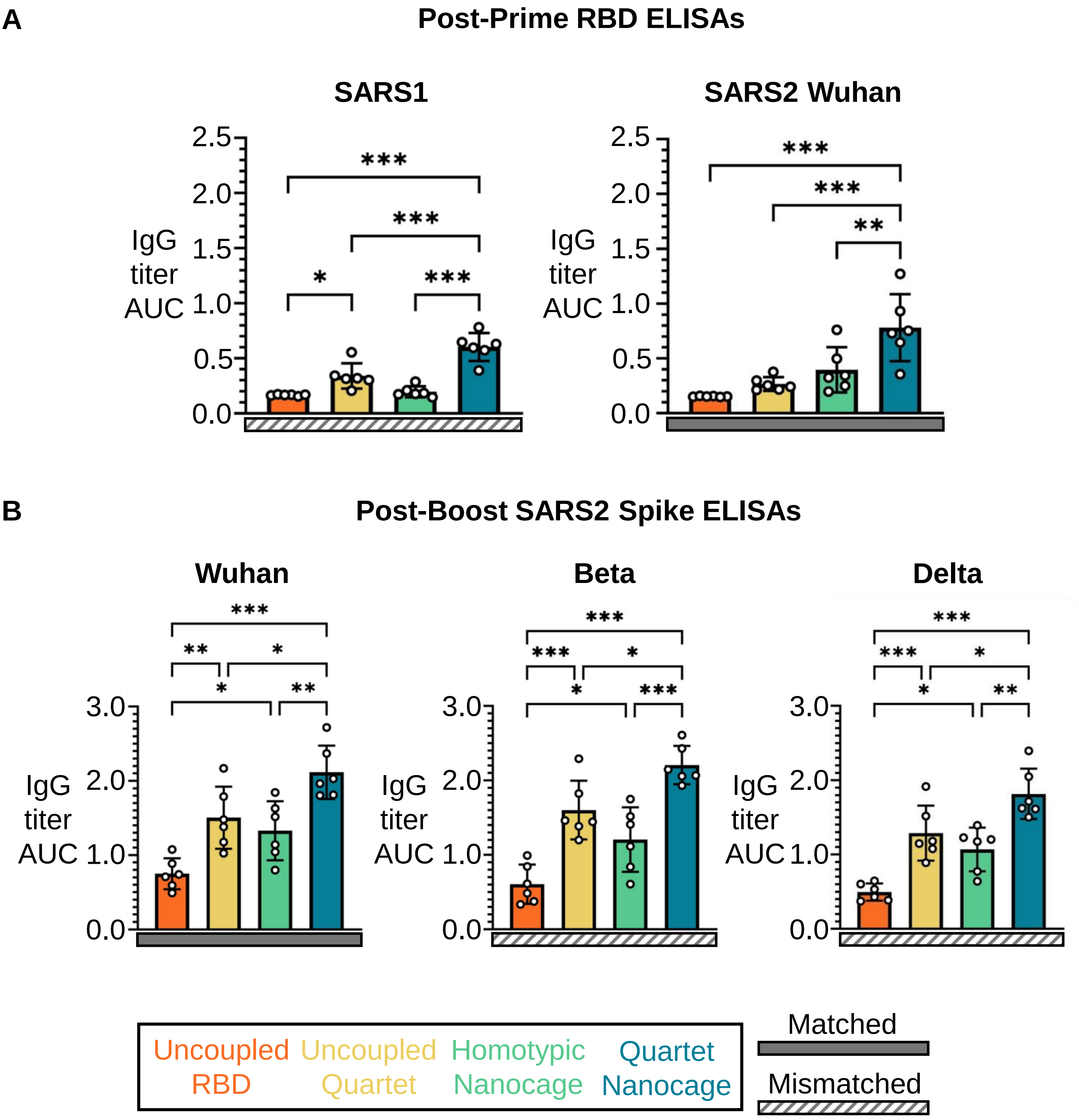

Supplementary Fig. 5. Further Breadth of Immune Response from Immunization with Quartet Nanocages. Binding data for serum IgG antibodies presented as area under the curve of a serial sera dilution. Sera samples are from mice immunized with uncoupled SARS2 Wuhan RBD (orange), Uncoupled Quartet (yellow), SARS2 Wuhan RBD coupled to SpyCatcher003-mi3 (Homotypic Nanocage, green), and Quartet Nanocage (blue) as outlined in Fig. 2. Solid gray rectangles under samples indicate the ELISA is against a component of that vaccine (matched), while striped rectangles indicate the ELISA is against an antigen absent in that vaccine (mismatched). Each dot represents serum from one animal. The mean is denoted by a bar, shown ± 1 s.d., n = 6. Significance was calculated with an ANOVA test using Tukey's post hoc test. * $p < 0.05$, ** $p < 0.01$, *** $p < 0.001$; other comparisons were non-significant. Graphs demonstrate the binding of (**A**) post-prime sera to RBDs and (**B**) post-boost sera to SARS2 variant Spike proteins.

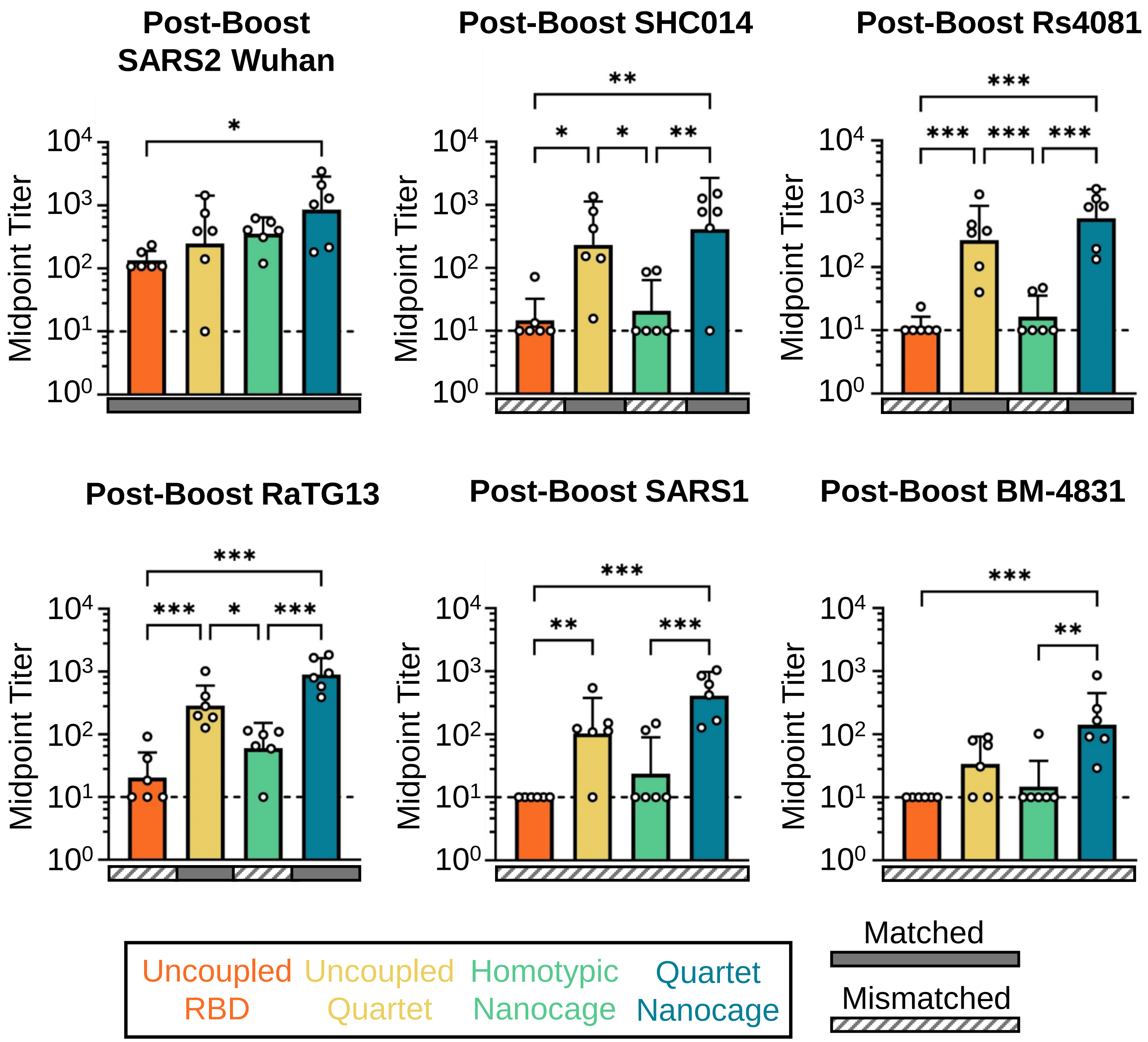

Supplementary Fig. 6. Midpoint Titers from Immunization with Quartet Nanocages. Binding data for serum IgG antibodies presented as midpoint titers, calculated from curve of a serial dilution of serum. Sera samples are from mice immunized with uncoupled SARS2 Wuhan RBD (orange), Uncoupled Quartet (yellow), SARS2 Wuhan RBD coupled to SpyCatcher003-mi3 (Homotypic Nanocage, green), and Quartet Nanocage (blue) as outlined in Fig. 2. Solid gray rectangles under samples indicate the ELISA is against a component of that vaccine (matched), while striped rectangles indicate the ELISA is against an antigen absent in that vaccine (mismatched). Each dot represents serum from one animal. The mean is denoted by a bar, shown + 1 s.d., n = 6. Significance was calculated with an ANOVA test, followed by Tukey's multiple comparison post hoc test of ID_{50} values converted to log_{10} scale. * $p < 0.05$, ** $p < 0.01$, *** $p < 0.001$; other comparisons were non-significant. Dashed horizontal lines represent the limit of detection.

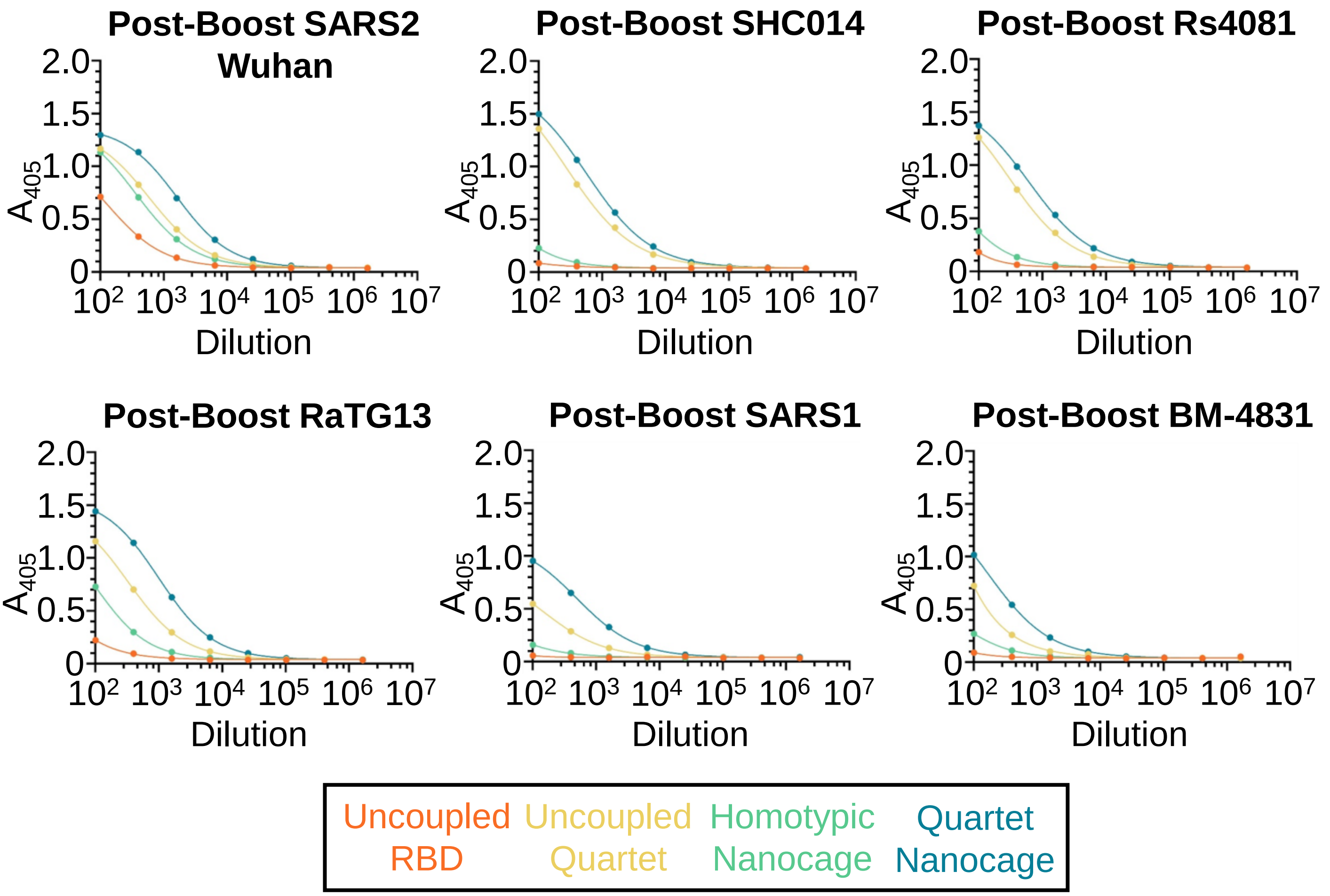

Supplementary Fig. 7. Serum binding curves from immunization with Quartet Nanocages. ELISA binding data are presented for a serial dilution of sera from mice immunized with uncoupled SARS2 Wuhan RBD (orange), Uncoupled Quartet (yellow), SARS2 Wuhan RBD coupled to SpyCatcher003-mi3 (Homotypic Nanocage, green), and Quartet Nanocage (blue) as outlined in Fig. 2. The mean absorbance (duplicate measurements for n=6 serum samples) for each immunization condition at each dilution is plotted. A curve is fit and plotted for each immunization condition.

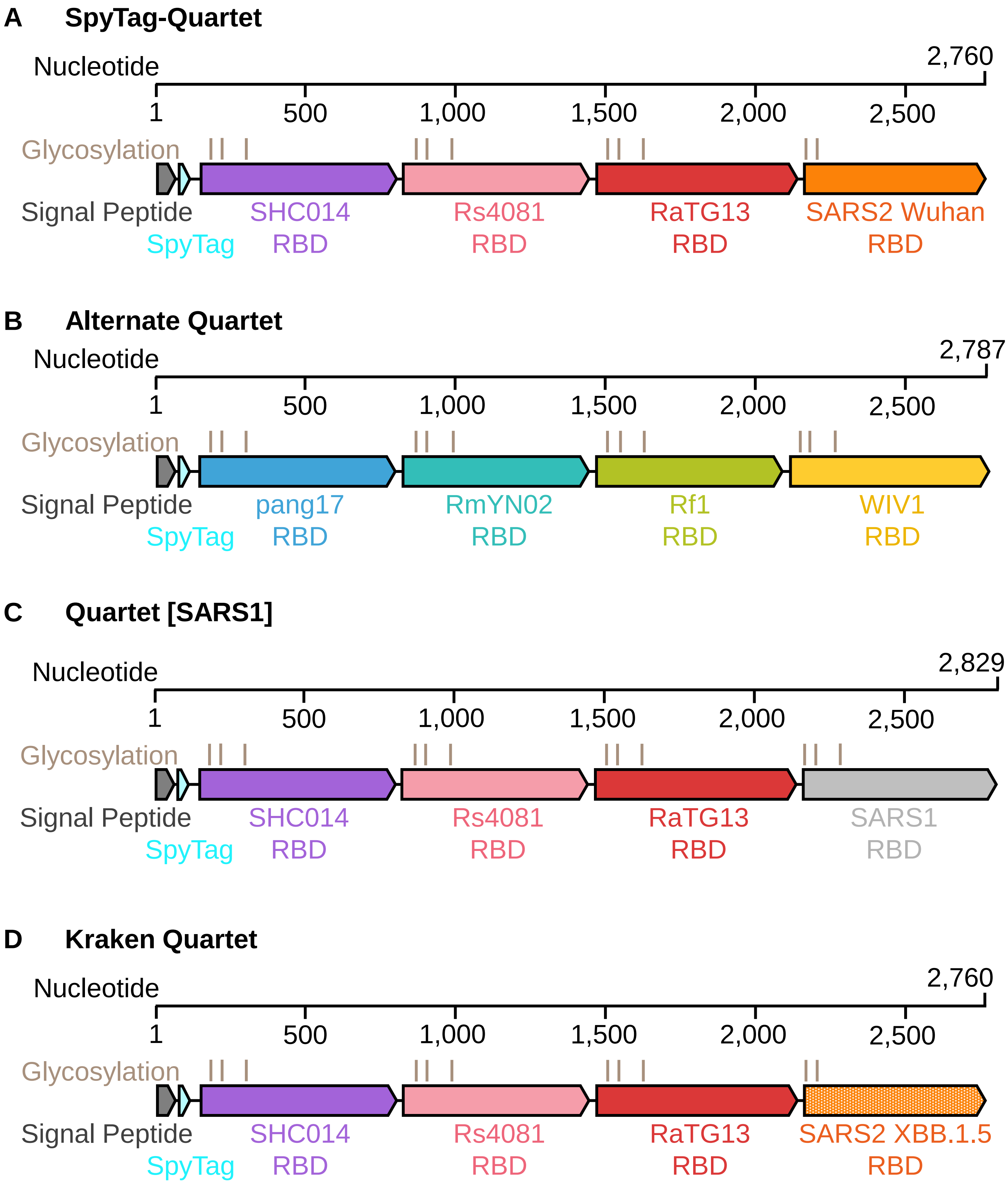

Supplementary Fig. 8. Schematic of Different Quartets. Genetic organization of (**A**) SpyTag-Quartet, (**B**) Alternate RBD Quartet, (**C**) Quartet [SARS1], and (**D**) Kraken Quartet (with SARS2 Omicron XBB.1.5)**.** These schematics indicate the virus origin of each RBD, predicted N-linked glycosylation sites, tag location, and nucleotide number.

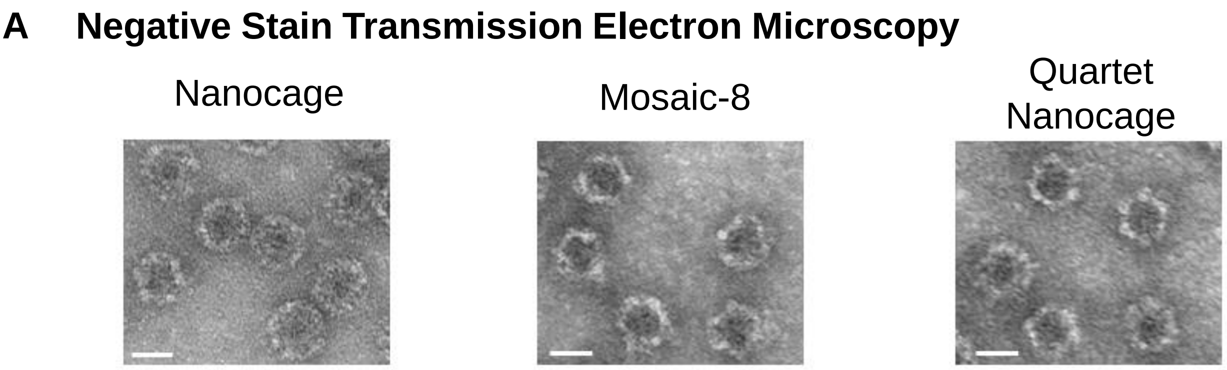

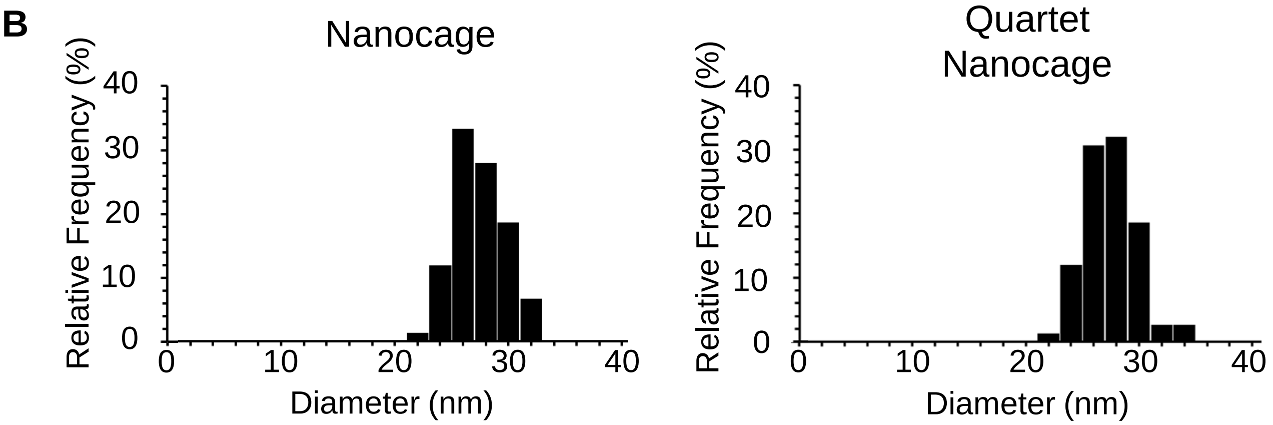

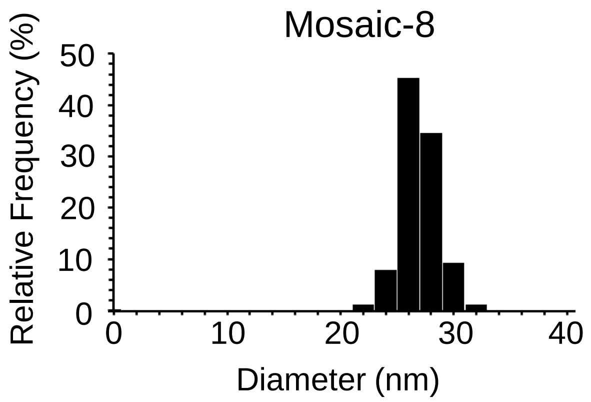

C

	**Diameter (nm)**
**Nanocage**	28.5 ± 2.2
**Mosaic-8**	27.9 ± 1.7
**Quartet Nanocage**	28.5 ± 2.3

Supplementary Fig. 9. Negative stain TEM of nanocage immunogens. (**A**) Representative negative stain TEM images of uncoupled Nanocage, Mosaic-8 nanoparticles, and Quartet Nanocages. Scale bar is 20 nm. (**B**) Size distribution of nanoparticles measured by TEM with 2 nm bin size (n = 75). (**C**) Table of the size distribution of different nanoparticles: mean ± 1 s.d. (n = 75).

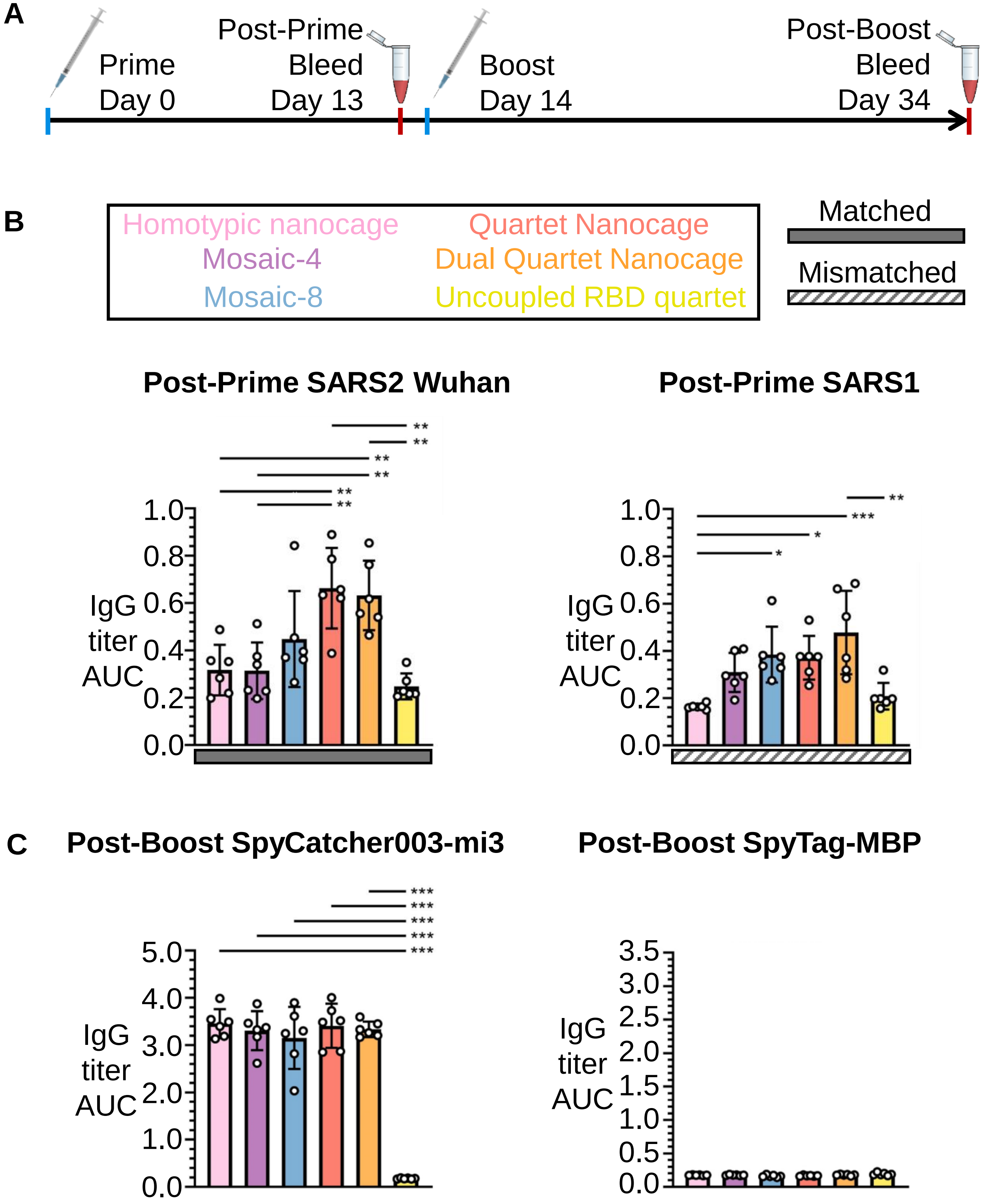

Supplementary Fig. 10. Breadth of antibody induction by Quartet and Mosaic Immunogens. (**A**) Summary of timeline for this set of immunizations with 0.02 nmol antigen per dose. **(B-C)** ELISA for serum IgG from mice immunized with the indicated immunogen. Each dot represents serum from one animal. (**B**) Post-prime response to SARS2 or SARS1. (**C**) Post-boost response to SpyCatcher003-mi3 or SpyTag-MBP. The mean is denoted by a bar, with error bars ± 1 s.d., n = 6. Significance was calculated with an ANOVA test using Tukey's post hoc test. No significance test was performed for SpyTag-MBP responses. * p < 0.05, ** p < 0.01, *** p < 0.001; other comparisons were non-significant.

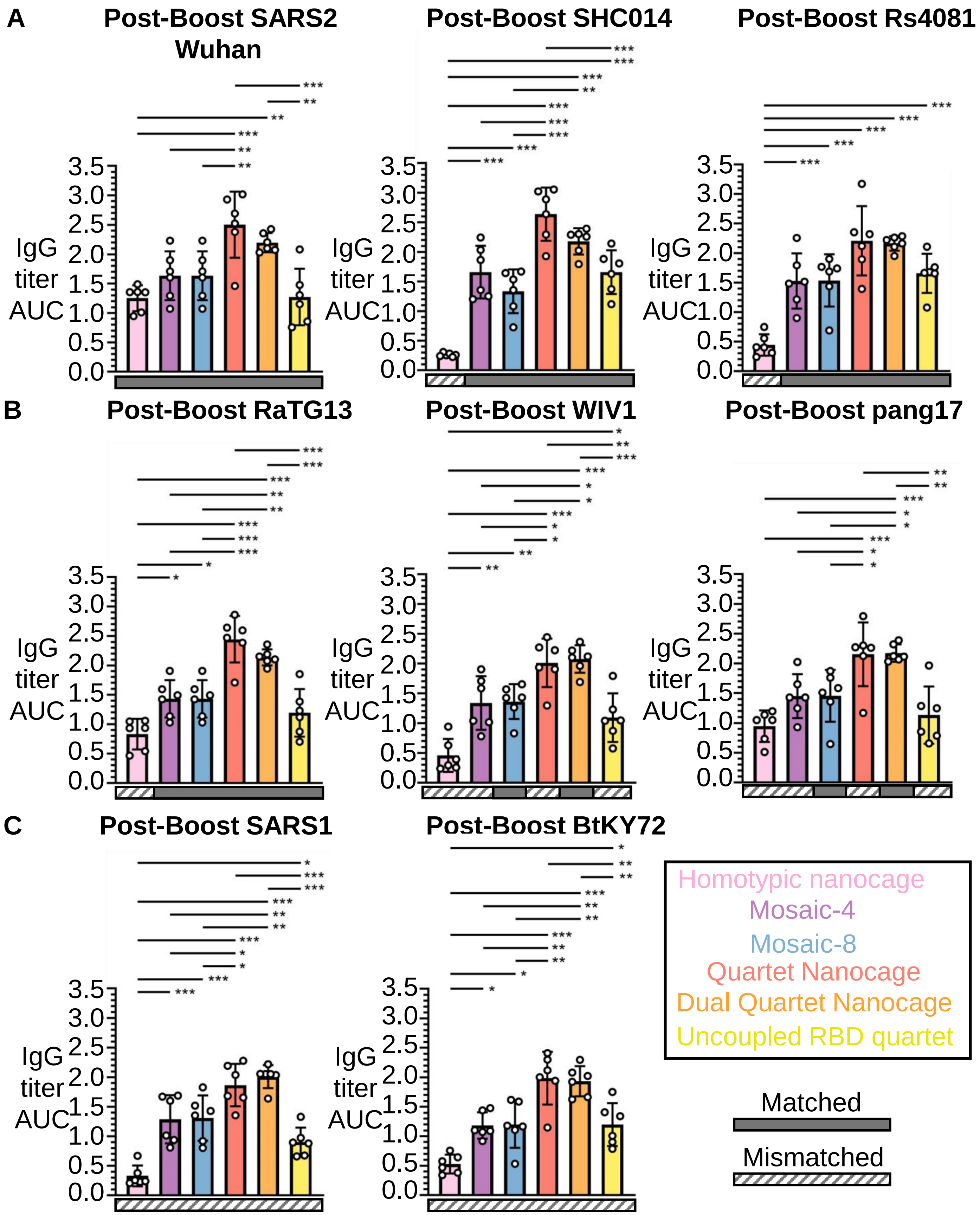

Supplementary Fig. 11. Further breadth of antibody induction by Quartet and Mosaic immunogens. ELISA for serum IgG from mice immunized with the indicated immunogen with 0.02 nmol antigen per dose presented as area under the curve of a serial sera dilution. Each dot represents serum from one animal. The mean is denoted by a bar, with error bars ± 1 s.d., n = 6. Significance was calculated with an ANOVA test using Tukey's post hoc test. * $p < 0.05$, ** $p < 0.01$, *** $p < 0.001$; other comparisons were non-significant. (**A**) Post-boost response to SARS2, SHC014 and Rs4081. (**B**) Post-boost response to RaTG13, WIV1 and pang17. (**C**) Post-boost response to SARS1 and BtKY72.

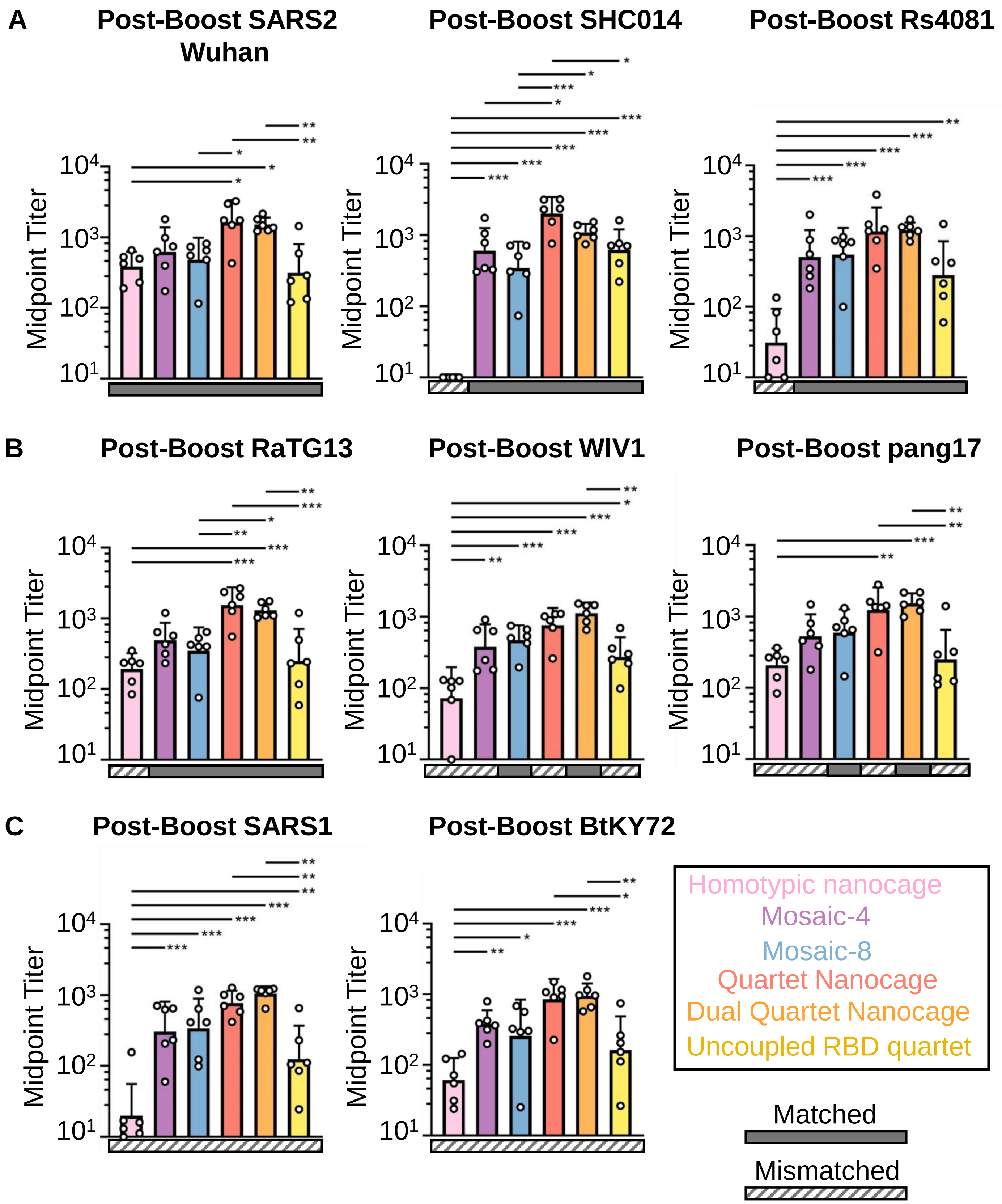

Supplementary Fig. 12 Midpoint titers for antibody induction by Quartet and Mosaic immunogens. ELISA for serum IgG from mice immunized with the indicated immunogen with 0.02 nmol antigen per dose presented as midpoint titers. Each dot represents serum from one animal. The mean is denoted by a bar, with error bars + 1 s.d., n = 6. Significance was calculated with an ANOVA test using Tukey's post hoc test. * $p < 0.05$, ** $p < 0.01$, *** $p < 0.001$; other comparisons were non-significant. (**A**) Post-boost response to SARS2, SHC014 and Rs4081. (**B**) Post-boost response to RaTG13, WIV1 and pang17. (**C**) Post-boost response to SARS1 and BtKY72.

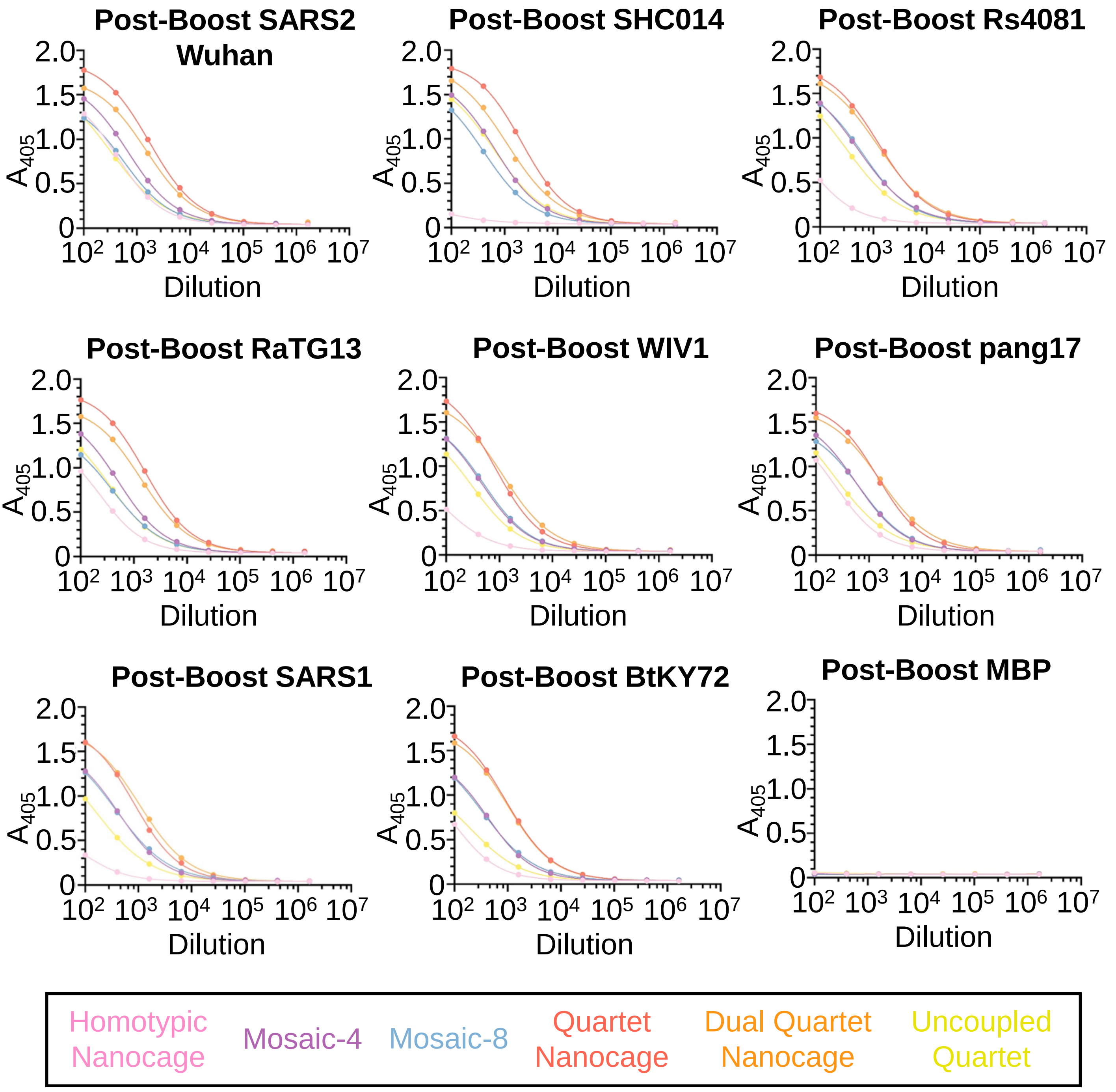

Supplementary Fig. 13. Serum binding curves for comparison of Quartet and Mosaic immunogens. ELISA binding data are presented for a serial dilution of sera from mice immunized with the indicated immunogen with 0.02 nmol antigen per dose presented as midpoint titers. The mean absorbance (duplicate measurements for n=6 serum samples) for each immunization condition at each dilution is plotted. A curve is fit and plotted for each immunization condition.

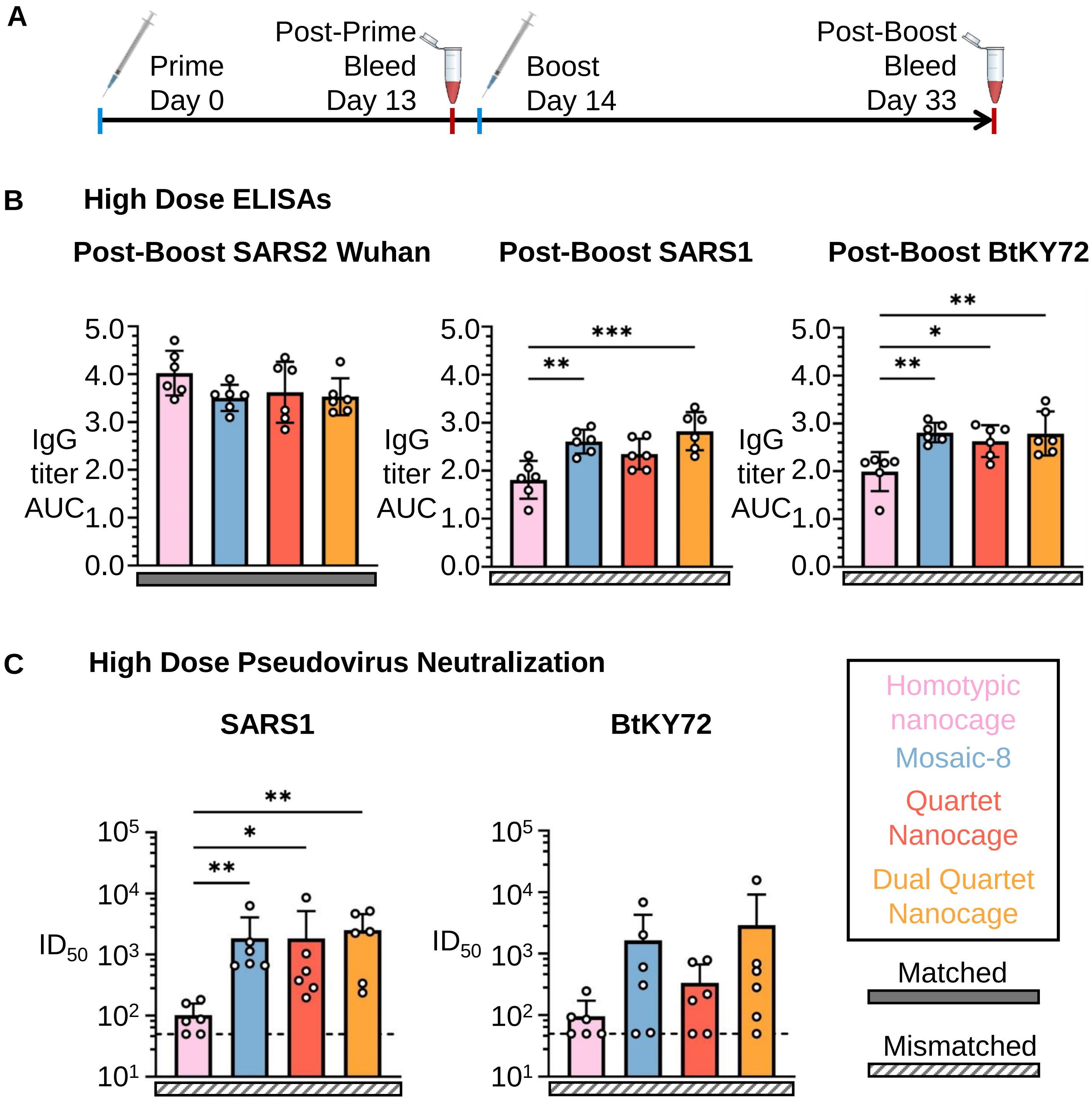

Supplementary Fig. 14. Immune response raised by higher dose of Quartet and Mosaic immunogens. This figure assesses antisera raised by immunizations with 0.2 nmol antigen. (**A**) Timeline for this set of immunizations. **(B)** ELISA for post-boost sera assessing IgG binding to SARS2, SARS1 and BtKY72 RBD is shown as the area under the curve (AUC) of a serial dilution. Each dot represents serum from one animal. The mean AUC is denoted by a bar, with error bars ± 1 s.d., n = 6. (**C**) Neutralization of SARS1 and BtKY72 (K493Y/T498W) pseudovirus by boosted mouse sera. Solid gray rectangles under samples indicate the ELISA is against a component of that vaccine (matched). Striped rectangles indicate the ELISA is against an antigen absent in that vaccine (mismatched). Dashed horizontal lines represent the limit of detection. The mean ID_{50} is denoted by a bar, with error bars + 1 s.d., n = 6. Significance was calculated with an ANOVA test, followed by Tukey's multiple comparison post hoc test of ID_{50} values converted to log_{10} scale. * $p < 0.05$, ** $p < 0.01$, *** $p < 0.001$; other comparisons were non-significant.

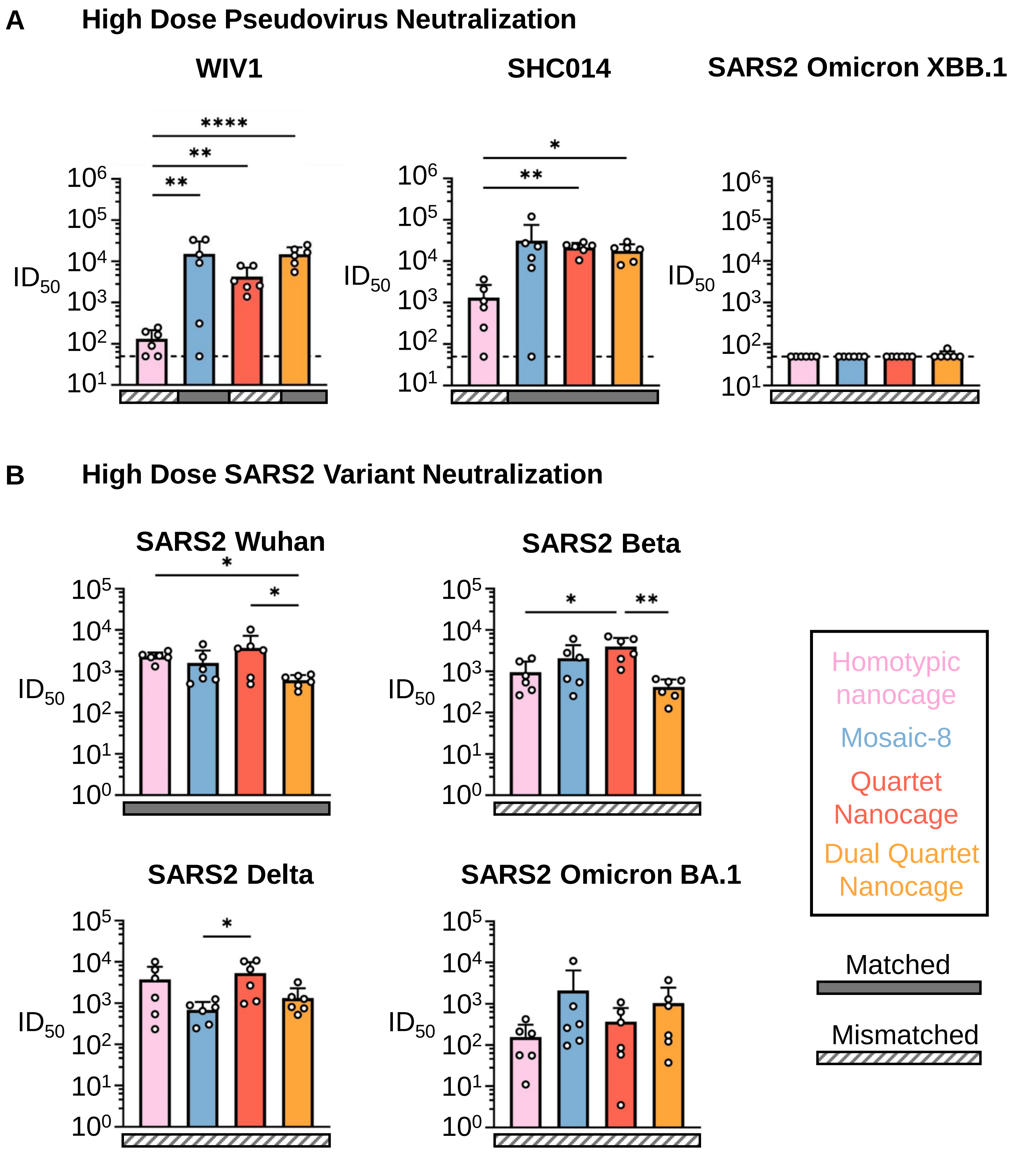

Supplementary Fig. 15. Pseudovirus neutralization by higher dose of Quartet and Mosaic immunogens. These figures assess antisera raised by immunizations with 0.2 nmol antigen, a 10-fold increase relative to prior immunizations. Solid gray rectangles under samples indicate the ELISA is against a component of that vaccine (matched). Striped rectangles indicate the ELISA is against an antigen absent in that vaccine (mismatched). Dashed horizontal lines represent the limit of detection. Each dot represents the response for one animal. Mean ID_{50} is denoted by a bar, with error bars + 1 s.d., n = 6. Significance was calculated with an ANOVA test, followed by Tukey's multiple comparison post hoc test of ID_{50} values converted to log_{10} scale. * $p < 0.05$, ** $p < 0.01$, *** $p < 0.001$; other comparisons were non-significant. (**A**) Neutralization of WIV1, SHC014 and SARS2 Omicron XBB.1 pseudoviruses. **(B)** Neutralization of the Wuhan, Beta, Delta and Omicron BA.1 SARS2 variant viruses.

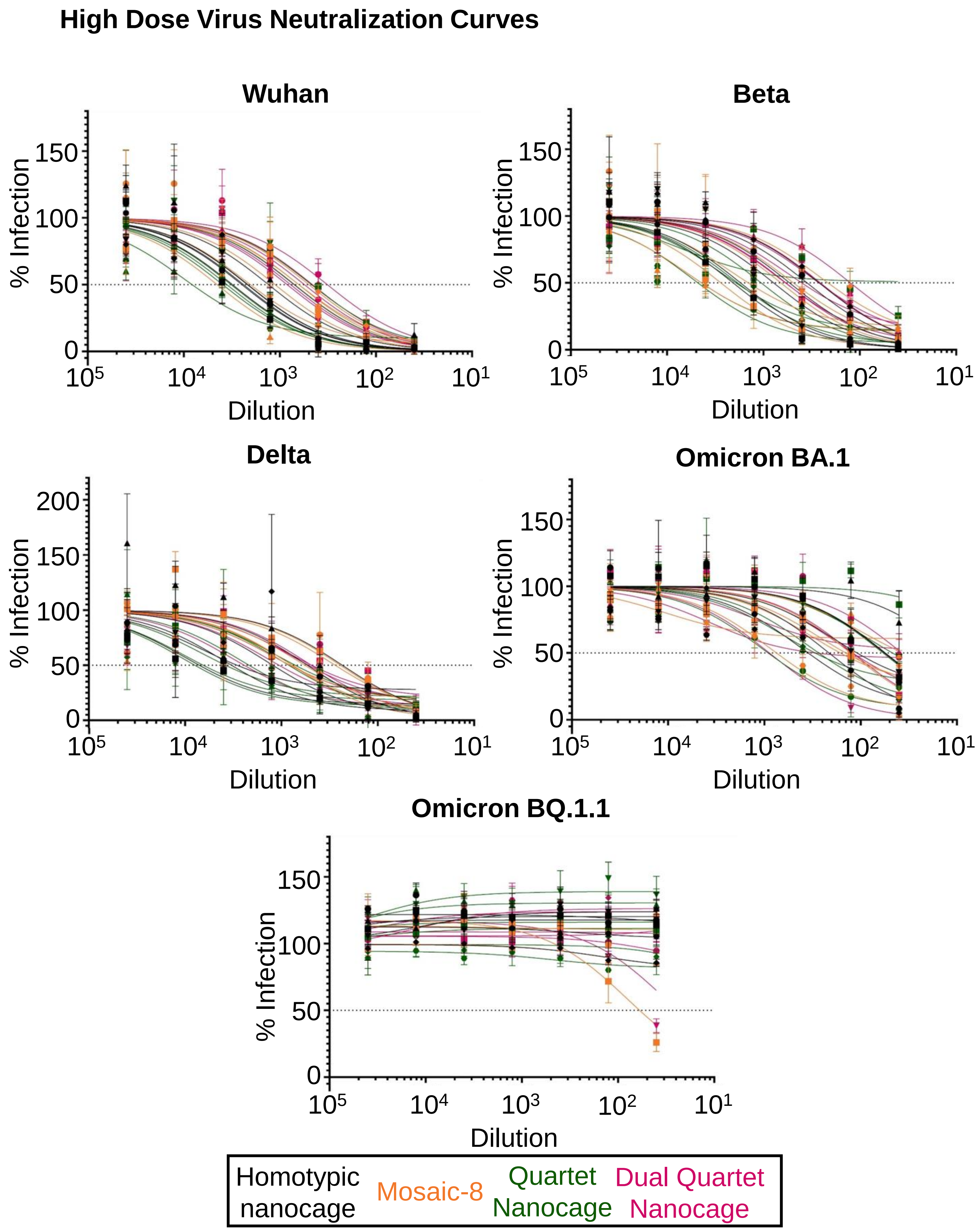

Supplementary Fig. 16. Authentic virus dose response curves. Neutralization of the Wuhan, Beta, Delta, Omicron BA.1, and Omicron BQ.1.1 SARS2 variants by antisera raised through immunizations with 0.2 nmol antigen. The percent of infection relative to a no-sera control (% Infection) was plotted relative to the dilution of sera. Each point is the mean of four replicates with error bars ± 1 s.d. and each curve is the response measured for a single animal (n = 6). These curves were used to determine the ID_{50} values plotted in Supplementary Fig. 15B. ID_{50} could not be calculated for Omicron BQ.1.1.

A Sarbecovirus pseudovirus neutralizations

	Homotypic Nanocage	Mosaic-8	Quartet Nanocage	Dual Quartet Nanocage
SARS1	2.0	3.1	2.8	3.2
BtKY72	1.9	2.6	2.3	2.6
SHC014	2.8	3.9	4.3	4.2
WIV1	2.0	3.6	3.5	4.1
SARS2 XBB.1	1.7	1.7	1.7	1.7

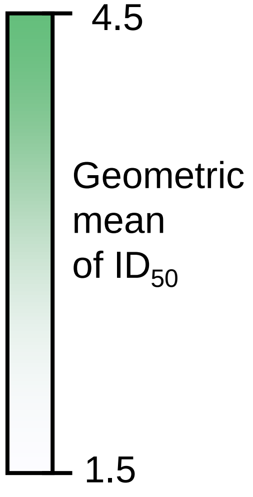

B SARS2 VOC neutralizations

	Homotypic Nanocage	Mosaic-8	Quartet Nanocage	Dual Quartet Nanocage
Wuhan	3.3	3.1	3.4	2.8
Beta	2.9	3.1	3.5	2.6
Delta	3.3	2.8	3.6	3.0
Omicron BA.1	2.0	2.7	2.1	2.6

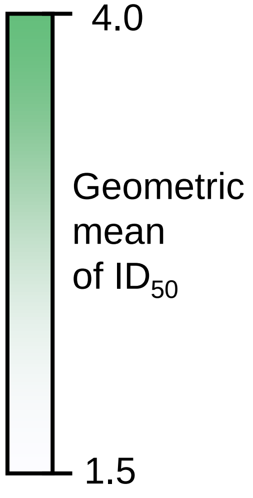

Supplementary Fig. 17. Summary of high dose virus and pseudovirus neutralizations. The geometric mean of ID_{50} for viral neutralization (n=6) with high dose immunogens. (**A**) Heat map of pseudovirus neutralization by sera raised through immunizations with 0.2 nmol of the specific antigen, summarizing data presented in Supplementary Figure 14 and 15. (**B**) Heat map of SARS2 VOC neutralization by sera raised through immunizations with 0.2 nmol of the specific antigen, summarizing data presented in Supplementary Figure 15.

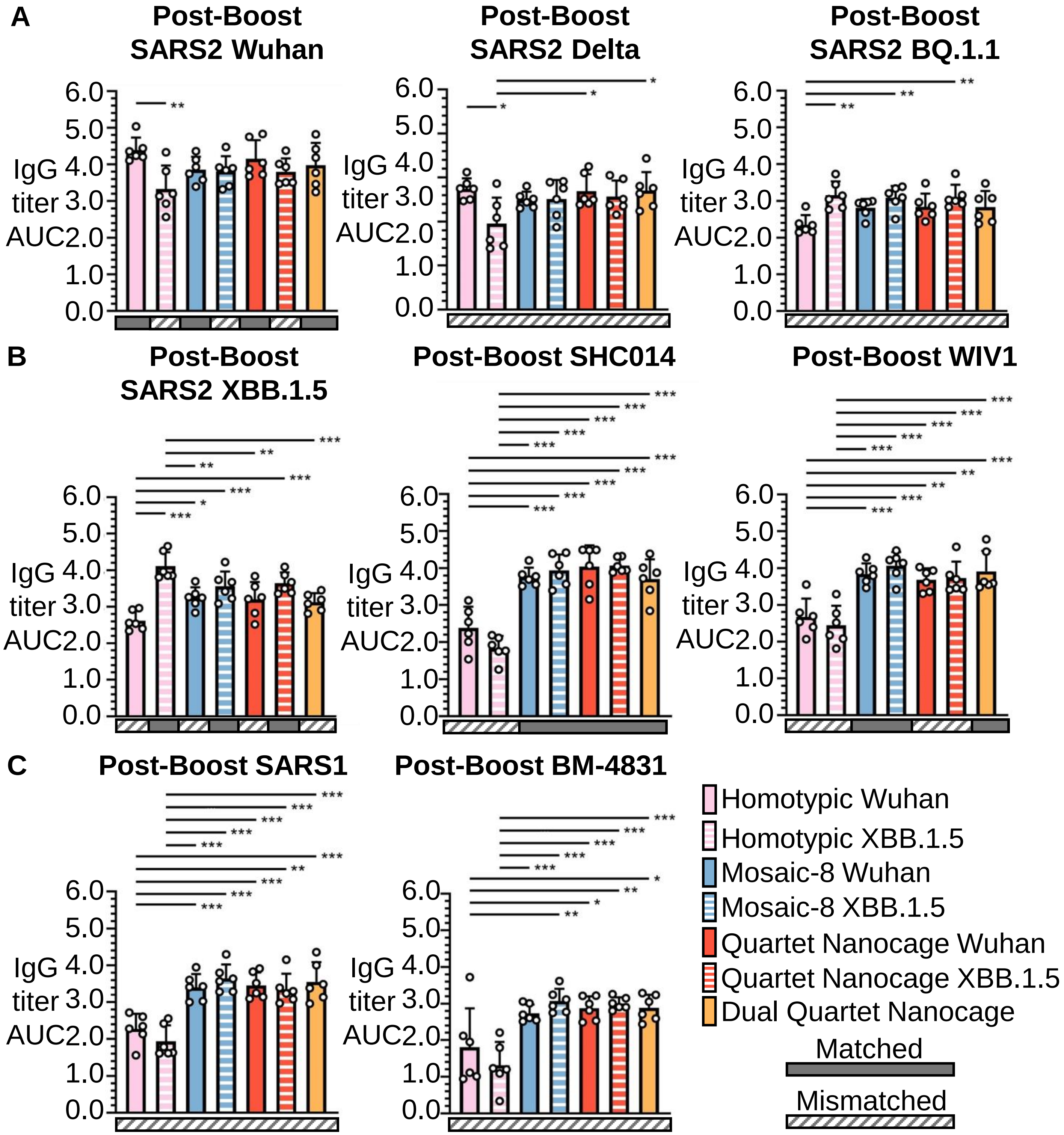

Supplementary Fig. 18. ELISA binding for immunogens with Wuhan or Kraken RBD. ELISA for serum IgG from mice immunized with the indicated immunogen with 0.2 nmol antigen per dose. Quartet Nanocage Wuhan is based on SpyTag-Quartet. Quartet Nanocage XBB.1.5 is based on Kraken Quartet. Dual Quartet Nanocage is based on SpyTag-Quartet and Alternate Quartet. Each dot represents serum from one animal. Solid gray rectangles under samples indicate the ELISA is against a component of that vaccine (matched), while diagonally striped rectangles indicate the ELISA is against an antigen absent in that vaccine (mismatched). The mean is denoted by a bar, with error bars ± 1 s.d., n = 6. Significance was calculated with an ANOVA test using Tukey's post hoc test. * $p < 0.05$, ** $p < 0.01$, *** $p < 0.001$; other comparisons were non-significant. Bars for immunogens containing SARS Wuhan are solid. Bars for immunogens containing SARS Omicron XBB.1.5 (Kraken) are striped. (**A**) Post-boost response to SARS2 Wuhan, SARS2 Delta, and SARS2 BQ.1.1. (**B**) Post-boost response to SARS2 XBB.1.5, SHC014 and WIV1. (**C**) Post-boost response to SARS1 and BM-4831.

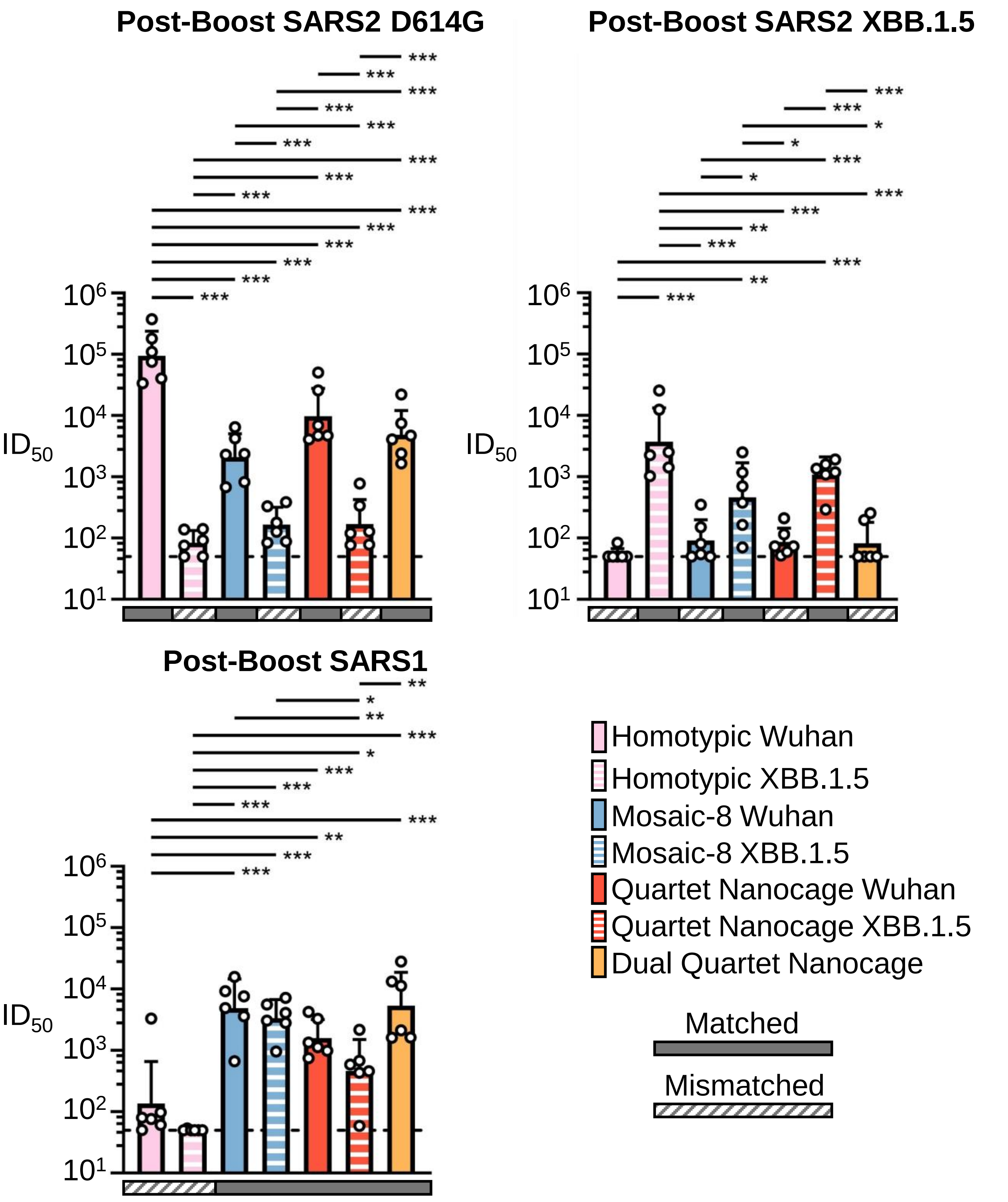

Supplementary Fig. 19. Pseudovirus neutralization for Quartet and Mosaic immunogens with Kraken RBD. Pseudovirus neutralization for serum IgG from mice immunized with the indicated immunogen with 0.2 nmol antigen per dose. Neutralization data are presented for SARS2 D614G (SARS2 Wuhan with a single point mutation), SARS2 XBB.1.5 and SARS1 Each dot represents serum from one animal. Solid gray rectangles under samples indicate the ELISA is against a component of that vaccine (matched), while diagonally striped rectangles indicate the ELISA is against an antigen absent in that vaccine (mismatched). The mean is denoted by a bar, with error bars + 1 s.d., with each dot representing the response for one animal (n = 6). Significance was calculated with an ANOVA test, followed by Tukey's multiple comparison post hoc test of ID_{50} values converted to log_{10} scale. * $p < 0.05$, ** $p < 0.01$, *** $p < 0.001$; other comparisons were non-significant. Bars for immunogens that contain SARS Wuhan are solid. Bars for immunogens that contain SARS Omicron XBB.1.5 (Kraken) are striped. Dashed horizontal lines represent the limit of detection.

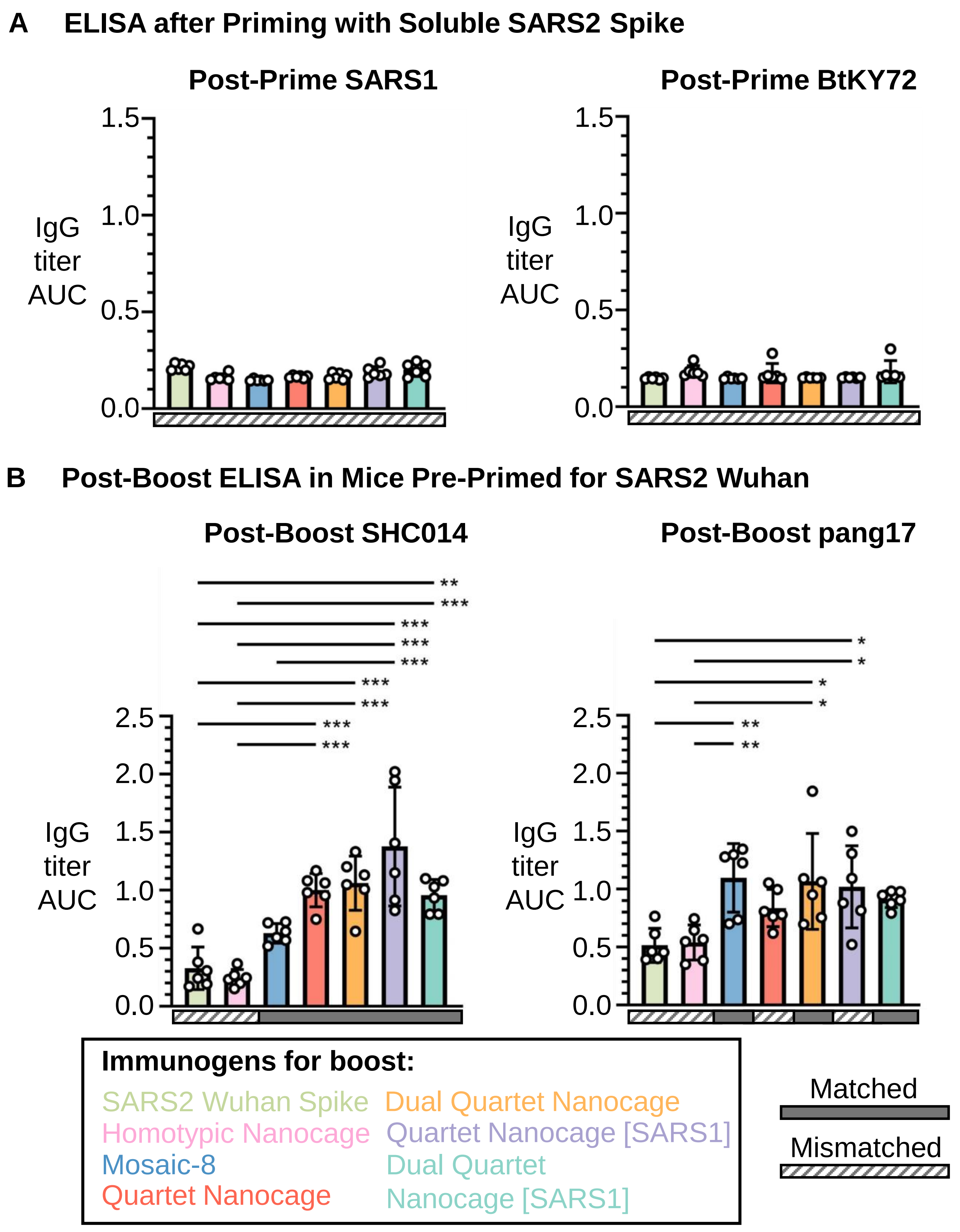

Supplementary Fig. 20. Further demonstration that Quartet immunization induces broad antibodies even after SARS2 Spike priming. (**A**) ELISA for serum IgG from mice immunized with a single dose of SARS2 Wuhan Spike protein, grouped by the second dose of 0.02 nmol antigen they will receive. No significance test was performed on post-prime samples. (**B**) ELISA for serum IgG, after mice immunized with a single dose of SARS2 Wuhan Spike protein were boosted with a variety of different antigens at 0.02 nmol per dose. Each dot represents serum from one animal. The mean is denoted by a bar, with error bars ± 1 s.d., n = 6. Significance was calculated with an ANOVA test using Tukey's post hoc test. * $p < 0.05$, ** $p < 0.01$, *** $p < 0.001$; other comparisons were non-significant.

A

Antibody	Class of RBD Epitope	Specificity
FI-3A	1	SARS2
C121	2	SARS2
FP-8A	1 or 2	SARS2
FD-5D	3	SARS2
FP-12A	4	SARS2
IY-2A	4	Broad
EY-6A	4	Broad
LCA60	n/a	MERS

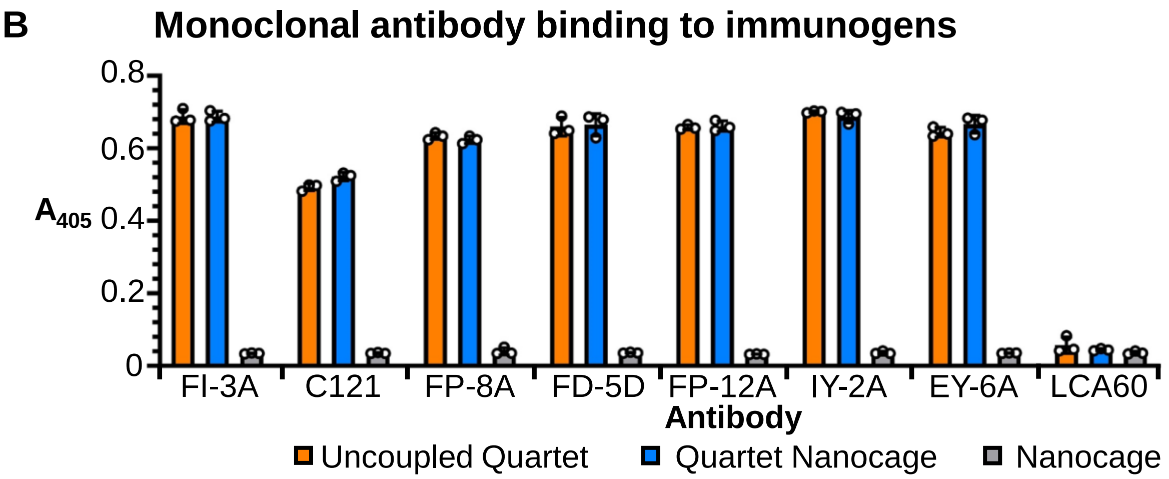

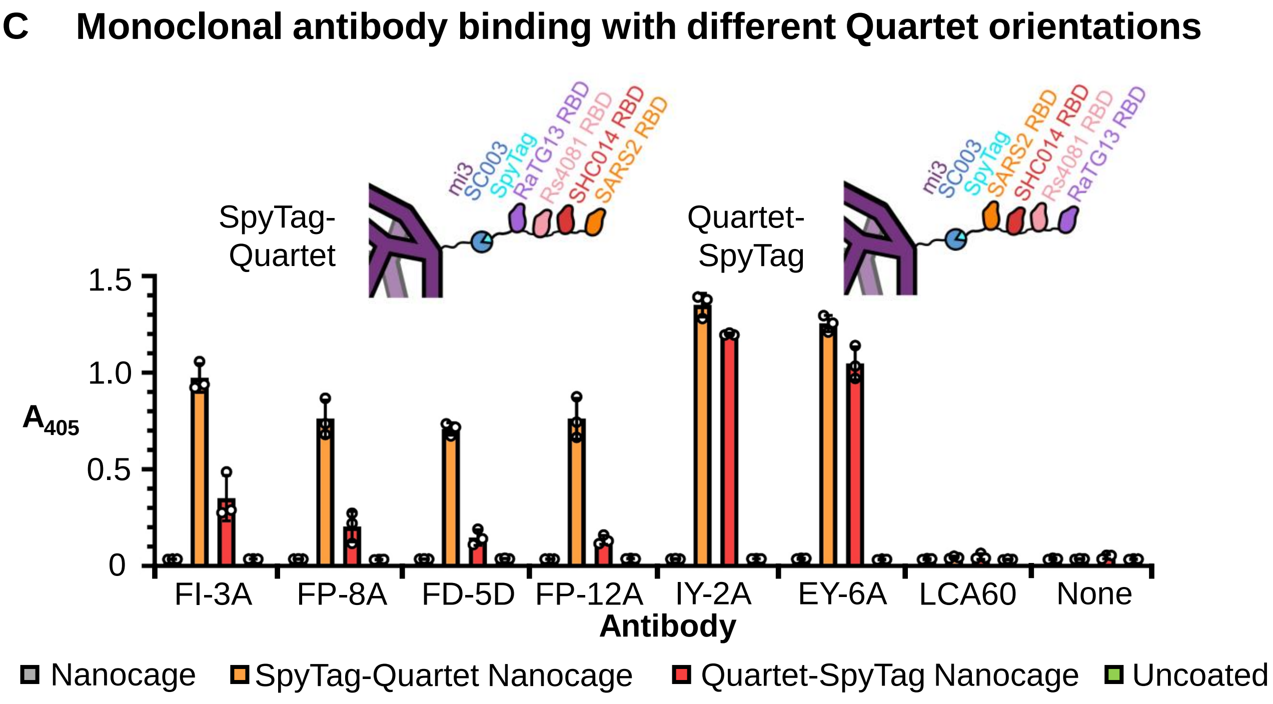

Supplementary Fig. 21. Monoclonal antibody binding to Quartet Nanocages. (**A**) Summary of monoclonal antibodies used in these experiments, with LCA60 as a negative control. (**B**) Comparison of monoclonal antibody binding to Quartet with and without coupling to SpyCatcher003-mi3. The mean absorbance for replicate wells (n=3) is denoted by a bar, with error bars ± 1 s.d.(**C**) Comparison of monoclonal antibody binding to Quartet coupled to SpyCatcher003-mi3 with different orientations. The mean absorbance for replicate wells (n=3) is denoted by a bar, with error bars ± 1 s.d.

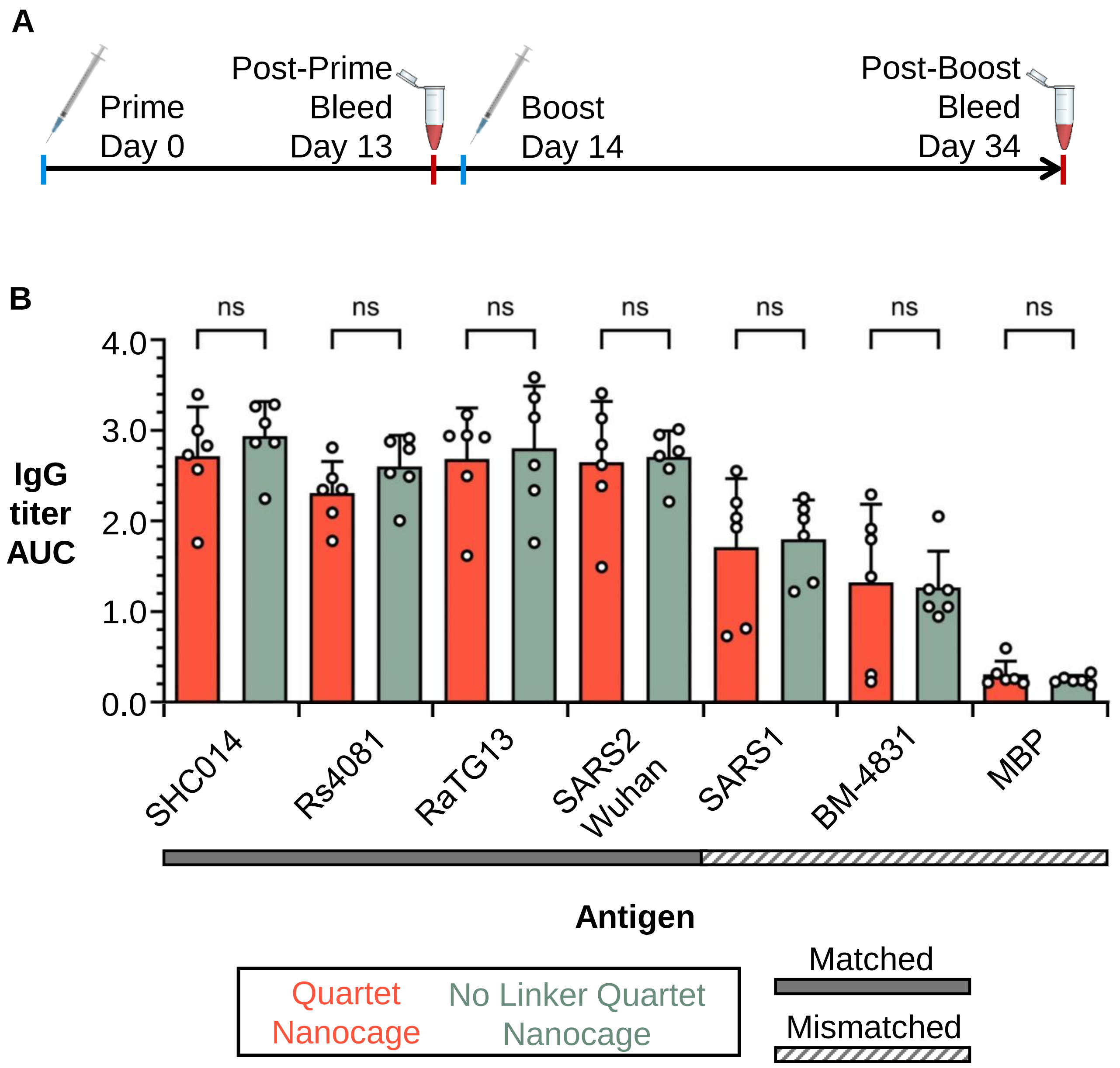

Supplementary Fig. 22. Immunogenicity for Quartets with or without linkers. Immunizations were performed with 0.02 nmol Quartet Nanocages from the conventional Quartet with flexible linkers between each RBD or No Linker Quartets with no linkers between RBDs. (**A**) Timeline for immunizations.(**B**) ELISA for serum IgG antibodies presented as area under the curve of a serial dilution of sera. Sera samples are from mice immunized using a conventional Quartet Nanocage (red) or a No Linker Quartet Nanocage (gray). Each dot represents serum from one animal. The mean is denoted by a bar, with error bars + 1 s.d., n = 6. ns means non-significant. Statistical comparisons were only made between responses to each antigen with or without linkers. Significance was calculated with an ANOVA test using Tukey's post hoc test. Solid gray rectangles under samples indicate the ELISA is against a component of that vaccine (matched), while diagonally striped rectangles indicate the ELISA is against an antigen absent in that vaccine (mismatched).

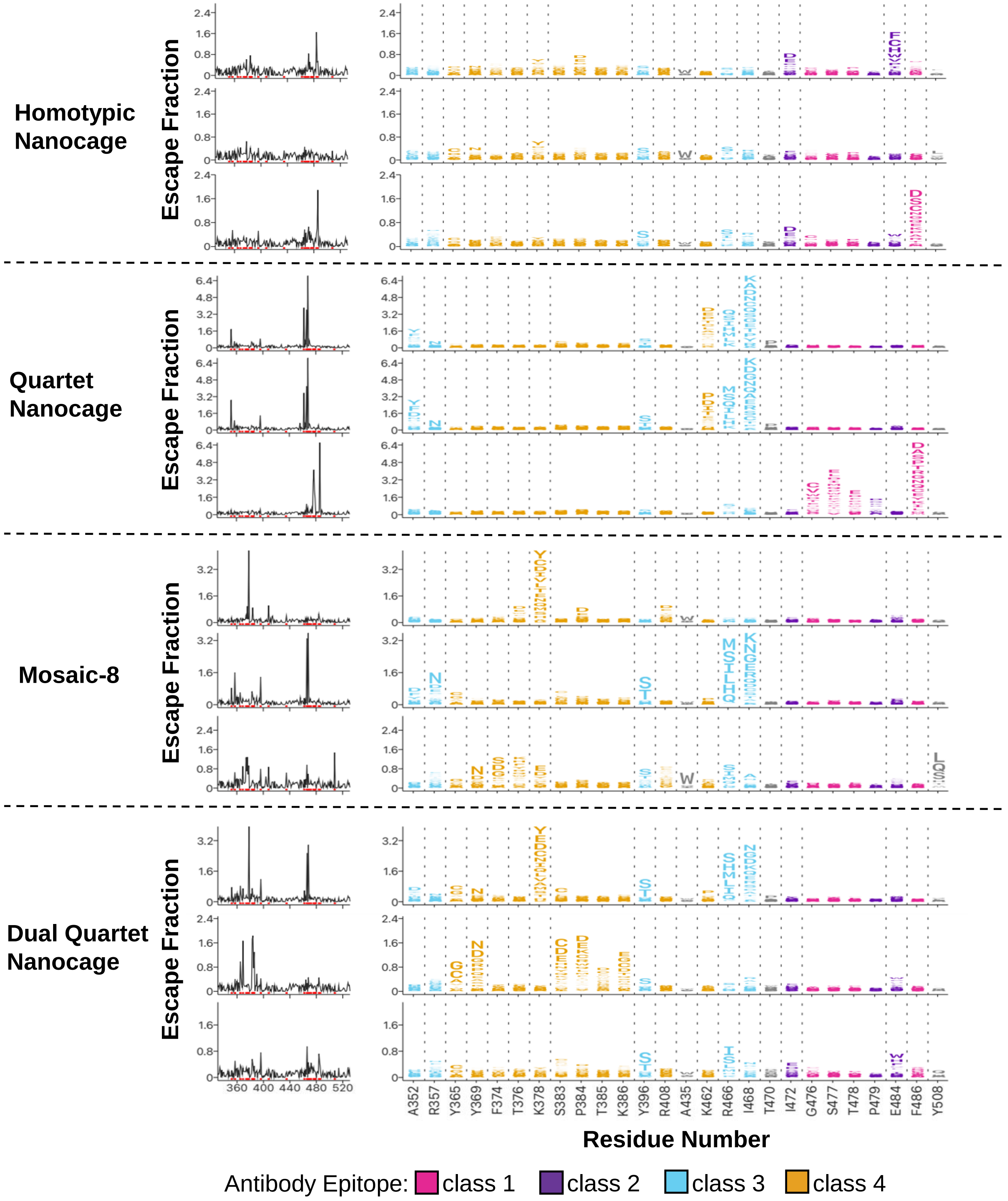

Supplementary Fig. 23. Antibody-escape maps for sera from immunization with Quartet and Mosaic antigens. Analysis by deep mutational scanning of sera of mice (n=3) primed and boosted with 0.2 nmol of each vaccine candidate. The line plots on the left visualize the sum of effects of all mutations at each RBD site on antibody binding; larger values indicate greater escape from antibody binding. Logo plots on the right show mutations that disrupt antibody binding. The height of each letter represents that mutation's escape fraction. Sites are colored by antibody epitope, while sites where some mutations introduce a potential N-linked glycosylation site sequon (NxS/T) are gray. The y-axis is scaled independently for each serum sample.

Supplementary Discussion

We gained additional insight using 10-fold higher antigen dose and the squalene-based adjuvant AddaVax to further enhance viral neutralization (Supplementary Figure 14A). Under these conditions Mosaic-8, Quartet Nanocage, and Dual Quartet Nanocage immunogens all raised a greater antibody binding response to SARS1 and BtKY72 RBD than Homotypic Nanocage. However, unlike lower-dose immunizations, there was no significant difference between the antibody titer raised by these different Mosaic and Quartet immunogens for any of the RBDs tested (Supplementary Figure 14B). Mosaic-8, Quartet Nanocage, and Dual Quartet Nanocage all elicited favorable neutralization of WIV1, SARS1, and SHC014 pseudovirus that were stronger than Homotypic Nanocage (Supplementary Figure 14C, Supplementary Figure 15, Supplementary Figure 17). Under these high-dose conditions there was no clear pattern for neutralization of SARS2 Wuhan, Beta, Delta, and Omicron BA.1 by the different immunogens (Supplementary Figure 15B, Supplementary Figure 16, Supplementary Figure 17). Antibody responses in mice can easily be saturated in a way that is hard to achieve in human clinical studies, thus obscuring important differences between vaccine candidates (*78*, *79*). Hence our low dose comparisons to Quartet Nanocages are likely to be most relevant for deciding on which candidates to take towards clinical development. No immunogen elicited substantial neutralization of SARS2 Omicron XBB.1 pseudovirus (Supplementary Figure 15A) or authentic Omicron BQ.1.1 virus using this immunization schedule (Supplementary Figure 16), which is consistent with the exceptional immune evasion found for Omicron variants (*80*).

Supplementary Discussion References

78. H. L. Davis, Novel vaccines and adjuvant systems: the utility of animal models for predicting immunogenicity in humans. *Hum Vaccin* **4**, 246–250 (2008).

79. S. P. Graham et al. Evaluation of the immunogenicity of prime-boost vaccination with the replication-deficient viral vectored COVID-19 vaccine candidate ChAdOx1 nCoV-19. *npj Vaccines* **5**, 1–6 (2020).

80. R. Uraki et al. Humoral immune evasion of the omicron subvariants BQ.1.1 and XBB. *The Lancet Infectious Diseases* **23**, 30–32 (2023).
