# Supplementary figures and images for: Proactive vaccination using multiviral Quartet Nanocages to elicit broad anti-coronavirus responses
Source: Nat Nanotechnol. 2024 May 6;19(8):1216–23. doi: 10.1038/s41565-024-01655-9 (PMC11329374; doi:10.1038/s41565-024-01655-9)

# Source Data for S3b

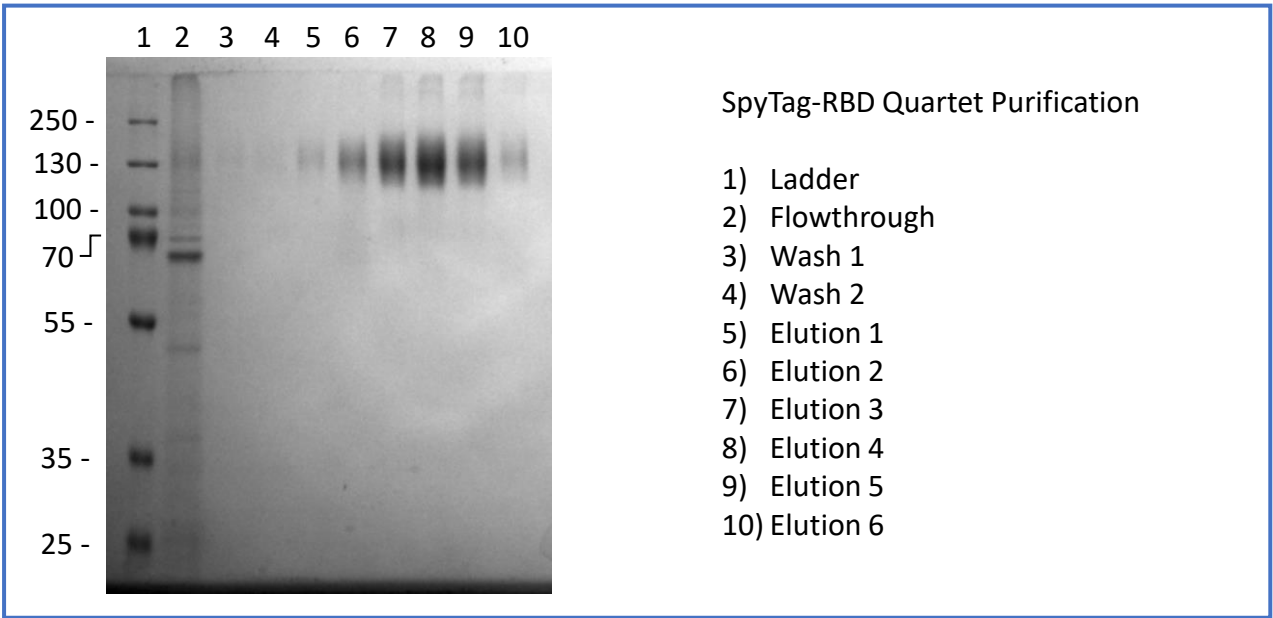

# Source Data for S3c

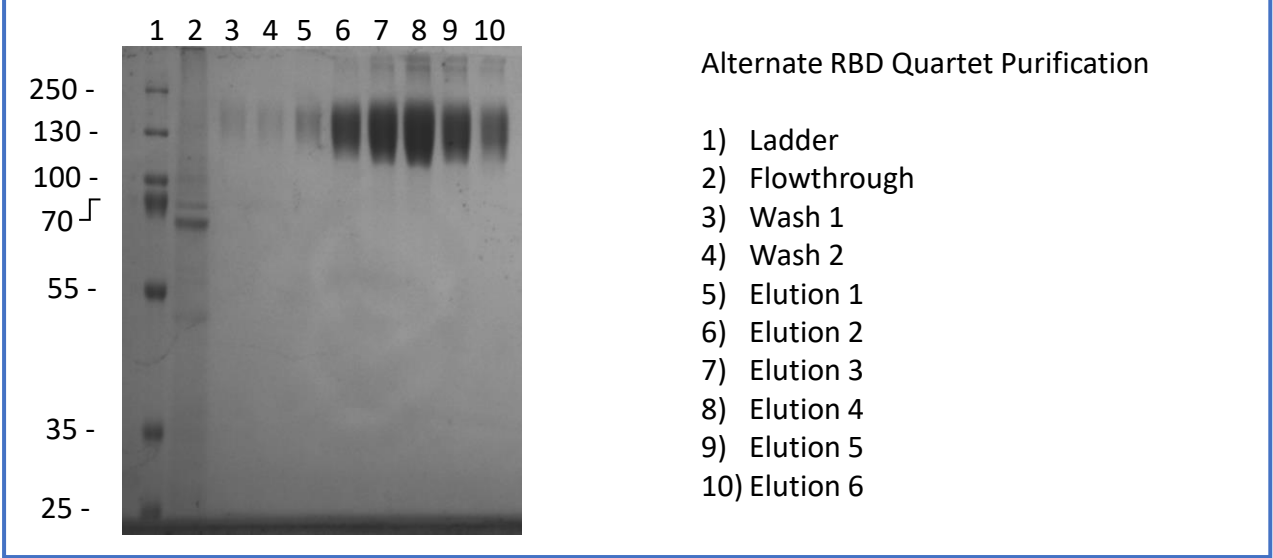

# Source Data for S3d

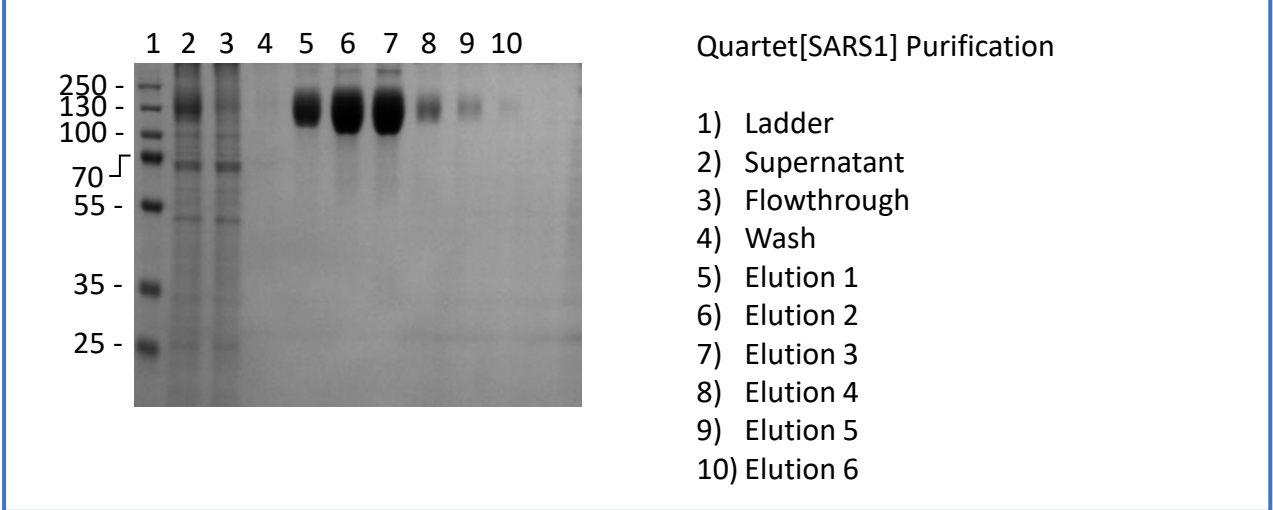

Supplement: Supplementary file 3 — Unprocessed gels. [file 41565_2024_1655_MOESM3_ESM.pdf]

## Source Data for 1d

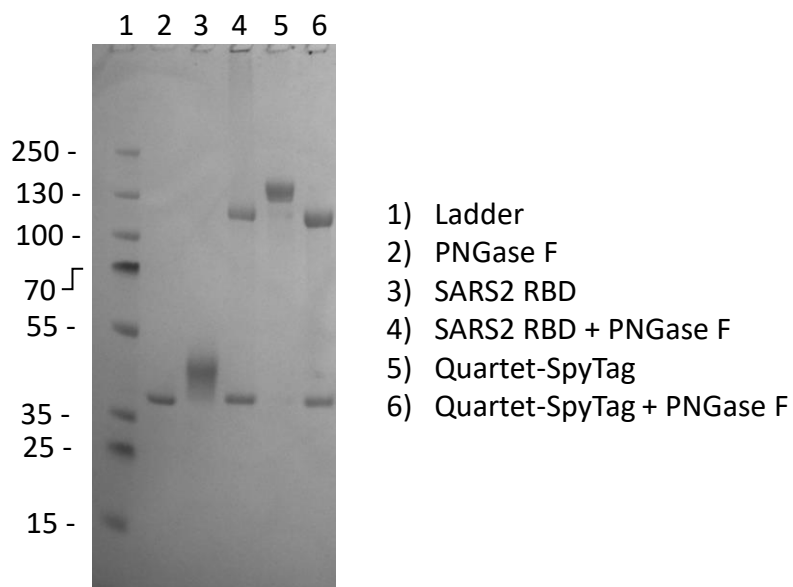

## Source Data for 1e

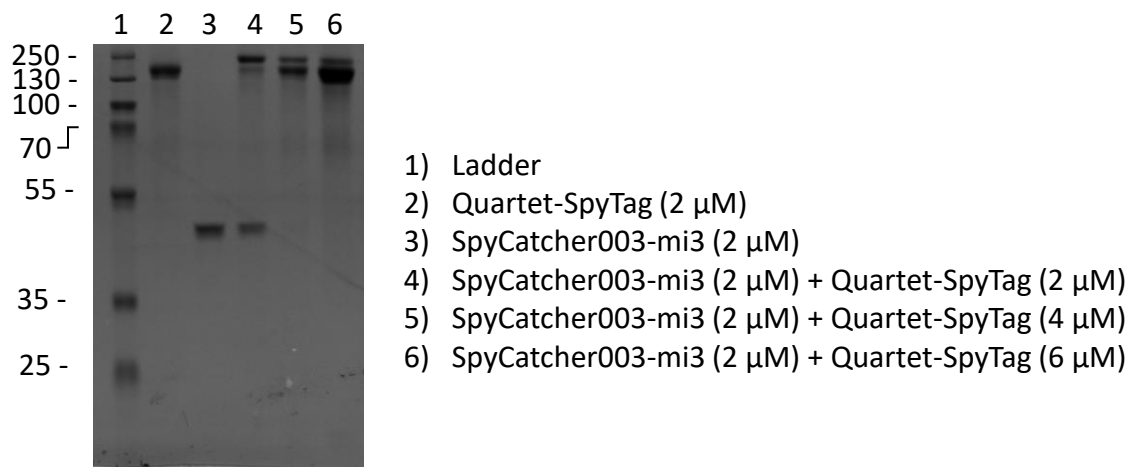

Supplement: Supplementary file 5 — Unprocessed gels. [file 41565_2024_1655_MOESM5_ESM.pdf]

Source Data for 2b

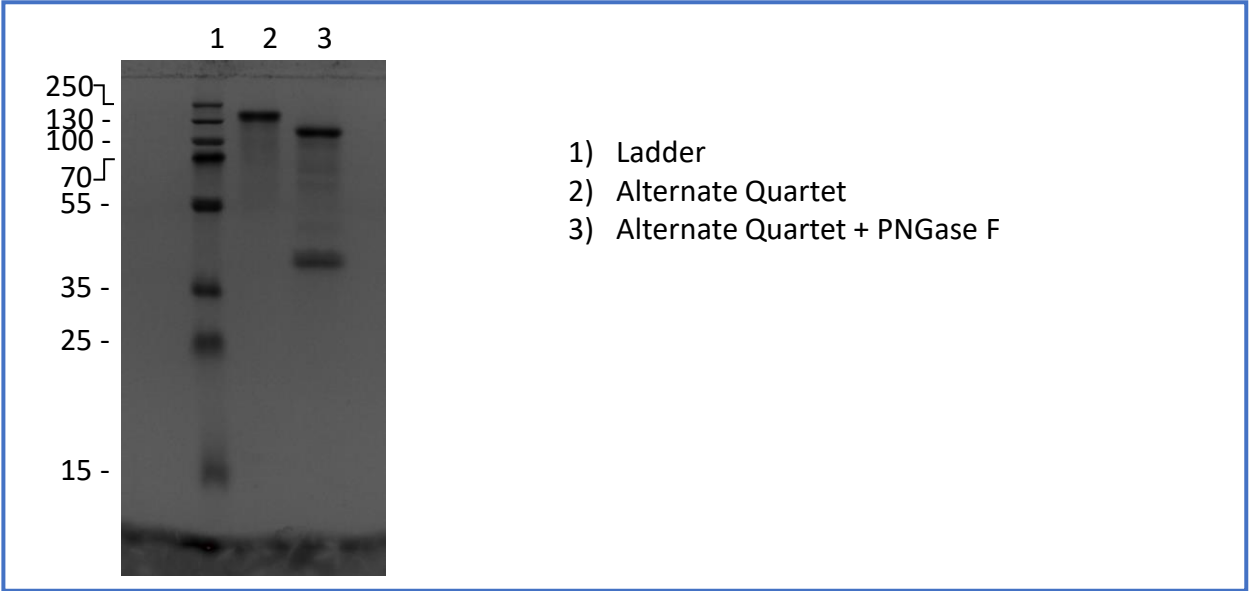

Supplement: Supplementary file 6 — Unprocessed gels. [file 41565_2024_1655_MOESM6_ESM.pdf]
